# Understanding structure-activity relationships in linear polymer photocatalysts for hydrogen evolution

**DOI:** 10.1038/s41467-018-07420-6

**Published:** 2018-11-23

**Authors:** Michael Sachs, Reiner Sebastian Sprick, Drew Pearce, Sam A. J. Hillman, Adriano Monti, Anne A. Y. Guilbert, Nick J. Brownbill, Stoichko Dimitrov, Xingyuan Shi, Frédéric Blanc, Martijn A. Zwijnenburg, Jenny Nelson, James R. Durrant, Andrew I. Cooper

**Affiliations:** 1 Department of Chemistry and Centre for Plastic Electronics, Imperial College London, Exhibition Road, London, SW7 2AZ UK; 2 Materials Innovation Factory, University of Liverpool, 51 Oxford Street, Liverpool, L7 3NY UK; 3 Department of Chemistry, University of Liverpool, Crown Street, Liverpool, L69 7ZD UK; 4 Department of Physics and Centre for Plastic Electronics, Imperial College London, Prince Consort Road, London, SW7 2AZ UK; 5 Department of Chemistry, University College London, 20 Gordon Street, London, WC1H 0AJ UK; 6 Department of Chemistry, Swansea University, Singleton Park, Swansea, SA2 8PP UK; 7 Stephenson Institute for Renewable Energy, University of Liverpool, Crown Street, Liverpool, L69 7ZD UK

## Abstract

Conjugated polymers have sparked much interest as photocatalysts for hydrogen production. However, beyond basic considerations such as spectral absorption, the factors that dictate their photocatalytic activity are poorly understood. Here we investigate a series of linear conjugated polymers with external quantum efficiencies for hydrogen production between 0.4 and 11.6%. We monitor the generation of the photoactive species from femtoseconds to seconds after light absorption using transient spectroscopy and correlate their yield with the measured photocatalytic activity. Experiments coupled with modeling suggest that the localization of water around the polymer chain due to the incorporation of sulfone groups into an otherwise hydrophobic backbone is crucial for charge generation. Calculations of solution redox potentials and charge transfer free energies demonstrate that electron transfer from the sacrificial donor becomes thermodynamically favored as a result of the more polar local environment, leading to the production of long-lived electrons in these amphiphilic polymers.

## Abstract (web summary)

While inorganic semiconductors are well-studied for their solar-to-fuel energy conversion abilities, organic materials receive far less attention. Here, authors prepare linear conjugated polymers as H_2_ evolution photocatalysts and rationalize photocatalytic activities with fundamental properties.

## Introduction

Direct photocatalytic water splitting has the potential to produce hydrogen as a clean fuel in a technologically simple way by using non-toxic, earth-abundant and stable photocatalysts^1–3^. While most photocatalysts are either inorganic^4^ or metal-organic materials^5^, polymeric organic photocatalysts have emerged recently as a new platform. For example, carbon nitrides^6^ can produce significant quantities of hydrogen when a sacrificial electron donor (SED) is used^7–10^ and are also active without sacrificial reagents when combined with metal co-catalysts or a second semiconductor^11–13^. The electronic properties of conjugated organic polymer photocatalysts^14–22^ can be fine-tuned by synthesis^18,23^. It is also possible to create photocatalytic polymer networks and frameworks with high levels of porosity^23–29^, and soluble organic polymer photocatalysts^16,17,20,30^ open up opportunities for large-area thin-film architectures and composite materials. The area of conjugated polymer photocatalysts has evolved rapidly in the past 3 years^20–22,24,27,31–36^, but it is fundamentally not well understood why different polymers show very different photocatalytic activities. Most attempts to rationalize activity trends link molecular structure to characteristics such as spectral absorption or the thermodynamic driving force for hydrogen production. In contrast, little is known about the underlying photophysics and surface processes of particulate polymer catalysts immersed in an aqueous medium. Typically, such systems use sacrificial agents to decouple hydrogen evolution from water oxidation, allowing the independent study of both half-reactions. In the presence of a SED the reaction sequence for hydrogen evolution is believed to involve photon absorption resulting in the formation of an excited electron–hole pair (exciton), exciton diffusion, hole transfer to the SED, and electron transfer to a proton, but there are no direct studies of this reaction sequence for polymer photocatalysts. This sequence is analogous to photoinduced charge transfer in organic photovoltaics, where photoexcitation of a polymer generates an exciton that dissociates after diffusion to the interface with a second material of different electron affinity (EA) or ionization potential (IP)^37^. Due to the low dielectric permittivity in organic semiconductors, the binding energy for these photogenerated carriers is normally too large for spontaneous dissociation at room temperature, but exciton diffusion to the polymer–water interface may allow photogenerated carriers to reach a reaction site for charge transfer to an electron or hole acceptor in the reaction medium. Such interfacial charge separation may be assisted by the higher relative permittivity of the aqueous medium, which helps to screen charges from each other^38^. The reaction mechanism has been proposed to proceed via anionic radicals^17^, but compelling evidence for the nature of the transient species and their impact on material performance is still lacking.

We investigate here a series of structurally related linear conjugated polymer photocatalysts with markedly different hydrogen evolution activities, including a homopolymer of dibenzo[*b*,*d*]thiophene sulfone, P10, with an external quantum efficiency (EQE, incident photon to hydrogen conversion yield) of 11.6% in the presence of a SED. Although we focus here on gaining a fundamental understanding of photocatalytic polymers rather than maximizing their activity, this is the highest EQE reported for any hydrogen-evolving polymeric photocatalyst outside carbon nitrides. We rationalize the differences in photocatalytic activity between the polymers in terms of differences in their light absorption, thermodynamic driving forces, excited state lifetimes, electronic structure, microstructure, and interfacial interactions.

## Results

### Polymer synthesis and characterization

P1, P7, and P10 were prepared by polymerizations of the respective dibromoarenes and diboronic acids or diboronic pinacol esters using Pd(0)-catalyzed Suzuki–Miyaura polycondensation at 150 °C in *N*,*N*-dimethylformamide in the presence of aqueous K_2_CO_3_ for 2 days, using methods similar to those that we reported previously (see Methods for details)^20^. Solid-state carbon-13 nuclear magnetic resonance (^13^C NMR) (Supplementary Figure 1, Supplementary Table 1) confirmed that polymerization had occurred and scanning electron microscopy (Supplementary Figure 2c) revealed flake-like primary particles ranging from 50 to 500 nm that aggregate into bigger particles ranging from 5 to 10 μm. The powder X-ray diffraction (PXRD) pattern of P10 (Supplementary Figure 2d) showed that the material is semi-crystalline.

**Fig. 1:**
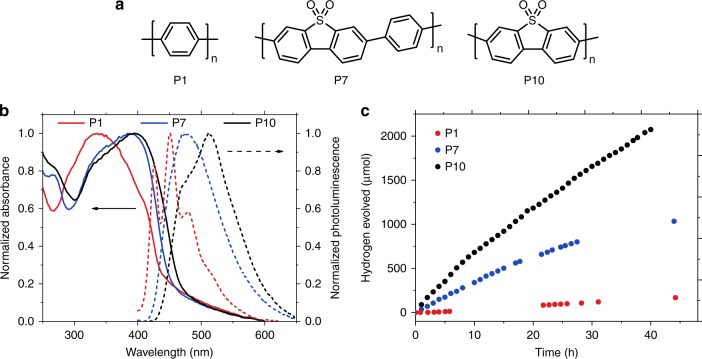
Polymer structures, optical properties, and hydrogen evolution experiments. **a** Chemical structures of polymers P1, P7, and P10. **b** UV–visible absorbance spectra and photoluminescence emission spectra at 345 nm excitation, acquired in aqueous suspension. **c** Time course for photocatalytic hydrogen evolution under visible light illumination (λ > 420 nm) using 25 mg photocatalyst in a 22.5 mL mixture consisting of equal volumes of H_2_O, MeOH, and TEA. A total of 2.07 mmol of hydrogen was evolved for P10, exceeding the amount of hydrogen present in P10 and therefore ruling out the polymer as the source of hydrogen

Figure 1b shows ultraviolet–visible (UV–Vis) absorbance spectra and photoluminescence emission spectra for the three polymers, as recorded in aqueous suspension, which suggests a slight red-shift of the absorption onset of P10 compared to both P1 and P7.

### Photocatalysis experiments

As shown in Fig. 1c, all three polymers yield steady hydrogen production under visible light illumination (λ > 420 nm) when suspended in a solvent mixture consisting of equal volumes of water, methanol, and triethylamine (TEA). TEA acts as a SED and methanol is used to reduce phase segregation between TEA and water^20,24,25^. While methanol is known to also act as a SED in some cases^6^, no activity has been observed in our case using either water or methanol alone and only limited activity (around 200 times lower than for TEA) was found for water–methanol mixtures. With a rate of 81.5 μmol h^−1^ for 25 mg photocatalyst (3260 μmol g^−1^ h^−1^) under λ > 420 nm illumination, P10 shows a significantly improved performance compared to P1 (1.6 μmol h^−1^) and also outperforms P7 (37.3 μmol h^−1^) by a factor of more than two^20^. When using a mixture of 5 vol. % TEA in water, the rates are reduced but remain significant (54.3 μmol h^−1^ for P10, Supplementary Figure 3) and the trend in activity between the polymers is reproduced. The EQE of P10 was estimated to be 11.6% at 420 nm using monochromatic light, whereas significantly lower values were obtained for P7 (7.2%) and P1 (0.4%) at the same wavelength^20^. P1 has a lower EQE compared to P10 even at 340 nm (4.1% vs. 8.9%, Supplementary Figure 4), which is close to the maximum absorption of P1. Similar results were obtained in photocatalytic experiments with a U-340 filter (transmissive in the range of 255–395 nm) gave rates of 8.2 µmol h^−1^ for P1, 19.6 µmol h^−1^ for P7, and 30.5 µmol h^−1^ for P10 (Supplementary Figure 5). Based on these experiments and the fact that differences in EQE are much higher than differences in absorption at the probe wavelength (Fig. 1b), we conclude that the extended visible light absorption of P10 is not the primary reason for its higher photocatalytic activity. While a long-term stability measurement of P10 over 40 consecutive hours showed a decrease in performance over time (by 33% after 10 h), no change in the UV–Vis and PL spectra or PXRD pattern is observed (Supplementary Figure 6).

All materials were tested as synthesized and no additional co-catalysts were added. However, significant levels of residual palladium were found entrained in all polymers (0.40 wt. % for P10, 0.38 wt. % for P7, 0.33 wt. % for P1), which has been proposed to have a catalytic effect^27,39,40^. Given that an activity plateau has previously been determined at palladium concentrations close to the ones reported here^27^, the small differences in Pd content between our polymers are not expected to account for their large differences in photocatalytic activity. In addition, a non-Suzuki P10 analog synthesized via oxidative Yamamoto coupling (P10Y, Supplementary Figure 7) exhibited a reduced but still significant hydrogen evolution rate of 30.0 μmol h^−1^, demonstrating hydrogen evolution activity in the absence of noble metals. Unlike most polymers that we have studied, P10 was also active under visible irradiation when suspended in a H_2_O/Na_2_S/Na_2_SO_3_ solution (19.8 μmol h^−1^, Supplementary Figure 8), which confirms that hydrogen is produced from water rather than by decomposition of the SED.

### Transient spectroscopy

**Fig. 2:**
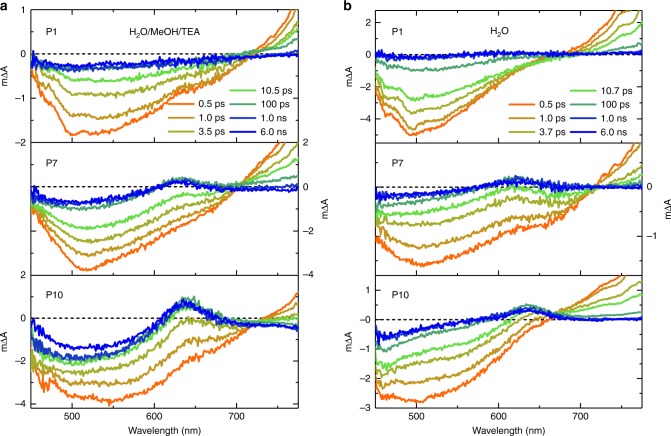
Spectral signatures of photogenerated reaction intermediates on the fs–ns timescale. Transient absorption spectra obtained from suspensions of P1, P7, and P10 (from top to bottom) in **a** a solvent mixture consisting of equal volumes of H_2_O, MeOH, and TEA and **b** in H_2_O. All data were obtained using an excitation wavelength of 355 nm and a fluence of 0.08 mJ cm^−2^

We next used transient absorption spectroscopy (TAS) to investigate the excited state dynamics of these polymers and the role of TEA in the photocatalytic process. Figure 2 shows transient spectra probed 0.5 ps to 6.0 ns after 355 nm excitation for suspensions of P1, P7, and P10, both in the reaction mixture H_2_O/MeOH/TEA and in pure H_2_O. Immediately after excitation, the transient spectra for all three polymers under both conditions consist of a broad negative signal in the 450–700 nm range and an excited state absorption above 700 nm. The excited state absorption peaks around 800–850 nm and tails off toward the infrared range (Supplementary Figure 9), which is in good agreement with reports of spectral signatures of singlet excitons in films of polymers that contain a similar fluorene backbone^41–43^. The negative transient signal between 450 and 700 nm overlaps spectrally with the polymer photoluminescence shown in Fig. 1b and it is therefore assigned primarily to stimulated emission from polymer excitons. For all three polymers, these two excitonic spectral features exhibit decays with a decay half time of ~10 ps, with P10 being slightly faster. This shows that higher photocatalytic activity is not related to longer exciton lifetime in this series (Supplementary Figure 10).

For P7 and P10, the spectra evolve over the first ~100 ps, at which point they exhibit a prominent new positive absorption feature around 630 nm with an appearance half-time of 1–2 ps (Supplementary Figure 11). As discussed below, slower timescales show a feature in the same spectral region that is assigned to an electron polaron on the polymer, that is, the active species for hydrogen evolution. Even on the faster timescale monitored here, the amplitude of this 630 nm feature in the reaction mixture correlates with the hydrogen evolution activity of the respective polymer, supporting an assignment to the active species. We note that a feature around 630 nm is also observed for P7 and P10 in H_2_O at ps–ns times (Fig. 2b). On this fast timescale, this feature may therefore be related to an exciton with increased charge transfer character, such as a polaron pair with electron and hole delocalized over adjacent chains, rather than an already fully separated charge. It is known that such polaron pairs and separated charges can give rise to very similar spectral signatures^44,45^.

**Fig. 3:**
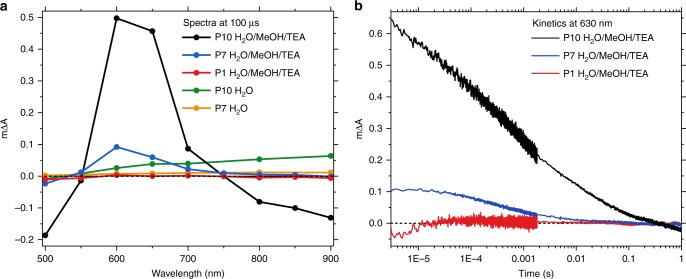
Spectral signatures of photogenerated reaction intermediates and their temporal evolution on the μs–s timescale. **a** Transient absorption spectra probed 100 µs after excitation for P10, P7, and P1 in the H_2_O/MeOH/TEA reaction mixture and for P10 and P7 in water alone. The spectrum for P1 in H_2_O is omitted as no appreciable signal was observed. **b** Transient kinetics probed at 630 nm for P10, P7, and P1 in the reaction mixture. All data were obtained at an excitation wavelength of 355 nm and a fluence of 0.32 mJ cm^−2^

To address the evolution of the 630 nm feature on longer timescales, we used μs–s transient absorption measurements. Transient spectra probed 100 µs after excitation using 355 nm light are shown in Fig. 3a. In the H_2_O/MeOH/TEA reaction mixture, a photoinduced absorption peak around 630 nm dominates the spectra for both P7 and P10, whereas this feature is absent for P1, in good agreement with the spectra at 6 ns in our ultrafast data (Fig. 2). This feature is only observed in the presence of TEA on this timescale, which supports its assignment to electrons on the polymer and discounts other possible origins such as triplet states. As mentioned, the resultant electron species are herein referred to as polarons. The amplitude of the 630 nm peak decreases in an oxygenated environment, which we assign to superoxide formation in competition with proton reduction, as is consistent with the lower hydrogen production yields in the presence of oxygen (Supplementary Figure 12). Measurements after addition of the electron scavenger methyl viologen to suspensions of P7 and P10 in reaction mixture indicate the presence of reducing intermediates (Supplementary Figure 13). Taken together, these observations support the hypothesis that electron polarons are formed upon irradiation via hole scavenging by TEA. This assignment is in good agreement with reported polaron signatures in polyfluorenes^41–43^ and is in line with the formation of long-lived electrons in inorganic photocatalysts upon addition of hole scavengers^46,47^ such as TEA.

From our ultrafast data, we conclude that the formation of the active species is largely complete within 100 ps (appearance half-time of 1–2 ps). On the μs and later timescale, the polaron evolution can be monitored by following the decay kinetics in the presence of TEA at 630 nm, as shown in Fig. 3b. At all times, the signal amplitude for P10 is significantly higher than for P7, which implies a larger concentration of polaron intermediates in suspension, consistent with the higher hydrogen evolution rate for P10. For both polymers, the 630 nm polaron absorption decays with an intensity-independent (Supplementary Figure 14) half-time of ~0.5 ms with respect to on the signal amplitude at 3 µs. Transient spectra of P10 in pure H_2_O and pure MeOH are almost identical, whereas a significant enhancement of the 630 nm feature is observed upon addition of TEA (Supplementary Figure 15). The signal amplitude at 630 nm increases up to a volume fraction of 33% in the reaction mixture for P7 and 25% for P10, as measured at a constant MeOH concentration (Supplementary Figure 16), which further demonstrates that the polaron yield is modulated by TEA rather than MeOH. This supports our interpretation that MeOH acts as a co-solvent rather than a hole scavenger, even when it is mixed with TEA, and is in good agreement with the low hydrogen evolution yields in H_2_O/MeOH (Supplementary Figure 17).

The sulfone group in P10 and P7 appears to play a key role in the process of charge generation in this polymer series because long-lived electron polarons, as assayed by the 630 nm absorption feature, are only observed for these two polymers. There is at least a qualitative correlation between photocatalytic activity and the appearance of the 630 nm absorption in our transient measurements. The absence of excited state absorption signals in P1 on longer (>ns) timescales suggests that excitons in this material do not lead to the production of useful charges, in good agreement with the low amount of hydrogen that P1 evolves under irradiation.

## Discussion

**Fig. 4:**
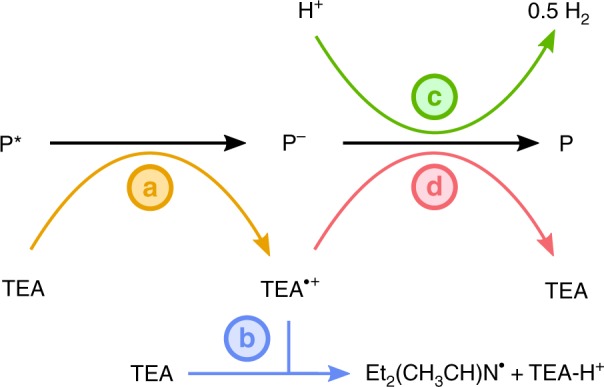
Photoinduced reaction scheme. First, a hole is transferred from the polymer exciton to TEA to yield the polymer electron polaron P^−^ and the radical cation TEA^•+^ **a**. Hence, the quantum efficiency of the TEA photooxidation determines the electron polaron yield, P^−^. The electron polaron population can then decay via electron transfer from the polymer to reduce a proton **c**; this stage would normally be rate limited by the availability of P^−^, not protons. The desired proton reduction process will be in kinetic competition with reactions such as the recombination of the electron polaron with TEA^•+^ radicals to regenerate the TEA **d** or the reduction of molecular oxygen. Assuming it survives recombination, the TEA^•+^ radical will evolve by deprotonation (where the proton is assumed to be accepted by a TEA molecule) **b**, hence reducing the amount of TEA^•+^ available for the back reaction **d**

To understand the mechanism of charge transfer on the timescale of the measurements, we consider the reaction sequence shown in Fig. 4 for the initial (<3 μs) timescales after photoexcitation. The slow (~0.5 ms) decay time of the TAS feature assigned to electron polarons shown in Fig. 3b is a lower limit for the timescale of proton reduction by P7 and P10 polarons and is consistent with the millisecond to early second timescales observed for proton reduction in other photocatalytic systems^48–50^. Our transient data demonstrate that hydrogen evolution activity is correlated with higher yields of electron polaron intermediates in suspension, and that the generation of these intermediates is correlated to the presence of sulfone groups in the polymer backbone. There are several possible explanations for the observed activities of the three polymers. First, different electronic properties might lead to different thermodynamic driving forces. Second, differences in polymer microstructure could influence the amount of polymer surface in contact with the reaction mixture. Third, different solvation properties could lead to a different organization of the solvent components at the polymer surface—something that has not been considered before for polymer photocatalysts. We address each of these possible explanations using a combination of molecular modeling and structural measurements.

First, we explore the size distribution of the particles in dispersion. Differences in polymer aggregate size, porosity, or surface roughness could affect the fraction of polymer volume available for charge transfer with the reaction mixture. Analysis of particle size distributions in purified water and in the reaction mixture by dynamic light scattering (DLS) showed that all three polymers exist as particles in the several hundred nm to micron range in water (Supplementary Table 2). P10 particles (635–660 nm) are smaller than P7 particles (840–990 nm), but P1 shows the smallest particle size (490–580 nm). Particle sizes in reaction mixture are larger for P7 and P10 than in water, but smaller for P1. The effect of liquid environment on particle size can be correlated with the relative polarity of the materials: the more polar, sulfone-based polymers disperse better in water than in the reaction mixture, while non-polar P1 disperses better in the less polar reaction mixture. Density functional theory (DFT) calculations confirm that the dibenzo[*b*,*d*]thiophene sulfone unit in P7 and P10 possesses a large static dipole of 5.7 D in vacuum pointing away from the sulfone, while the phenylene monomer has a zero dipole moment (Supplementary Figure 18). The hydrophilic nature of the sulfone group is supported by the lower contact angles of water droplets on P10 (59°) compared with P7 (67°) and P1 (88°) (Table 1 and Supplementary Figure 19). The DLS measurements show that all particle sizes are large relative to the likely diffusion length of excitons in these polymers, as exciton diffusion lengths in conjugated polymers typically range from a few nm to 10 nm in solid films^51–53^. Given the low surface area of these particles (Supplementary Figure 20), it seems likely that the majority of the excitons would be generated more than an exciton diffusion length away from the polymer–solvent interface. The observed exciton lifetime is rather insensitive to the presence of TEA (Supplementary Figure 10), supporting the idea that most photogenerated excitons recombine without charge transfer, even in the best-performing system, P10. While some degree of swelling might occur when the dry polymers are immersed in the reaction mixture, swelling effects are unlikely to entirely account for activity differences as large as the ones observed here given the relatively large non-porous particles. Thus, substantial further improvements in photocatalytic activity might be possible with control of the polymer microstructure.

**Table 1:** Polymer characterization and hydrogen evolution activity

Polymer	Optical gap (eV)	Contact angle vs. H_2_O (°)	HER >420 nm H_2_O/MeOH/TEA (μmol h^−1^)	EQE 420 nm (%)
P1	2.76	88 (±2.9)	1.6 (±0.1)	0.4 (±0.1)
P7	2.73	67 (±1.7)	37.3 (±0.8)	7.2 (±0.3)
P10	2.62	59 (±0.8)	81.5 (±4.1)	11.6 (±0.5)

Optical gap as calculated from the onset of the absorption spectrum in suspension; contact angle as measured on the surface of pressed pellets of the polymer; HER from 25 mg polymer suspended in a 22.5 mL mixture consisting of equal volumes of H_2_O, MeOH, and TEA;^20^ EQE determined using a 420 nm LED.

*HER* hydrogen evolution rate, *EQE* external quantum efficiency

**Fig. 5:**
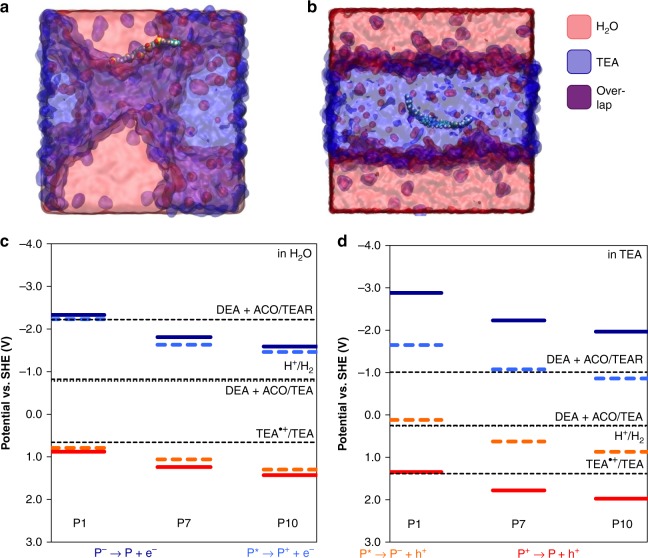
Molecular dynamics simulations and energy level calculations in water and mixed water/TEA environments. **a**, **b** Snapshots of atomistic molecular dynamics simulations of oligomers of **a** the polar polymer P10 and **b** a non-polar fluorene polymer, as a model for P1, both in a mixture of TEA (blue) and water (red). While the non-polar fluorene polymer hides in the TEA phase, the amphiphilic P10 polymer resides in a domain containing both TEA and water, typically close to the TEA–water interface. **c**, **d** Calculated ionization potential (IP, red solid line) and electron affinity (EA, blue solid line) for ground-state oligomers of P1, P7, and P10 together with the exciton electron affinity (EA*, orange dashed line) and ionization potential (IP*, blue dashed line), in comparison with the potentials for proton reduction (H_2_/H^+^), overall TEA oxidation (DEA+ACO/TEA), the first TEA oxidation step (TEA^•+^/TEA) and the second oxidation step of the TEA oxidation product (DEA+ACO/TEAR), using a **c** water or **d** TEA solvent environment

To address the microscopic interactions between polymer and reaction mixture, we simulated the polymer–liquid system using molecular dynamics (MD) simulation (see Methods and Supplementary Methods for details). Simulated equilibrated mixtures of water, or water and methanol, with TEA show phase-segregated TEA domains (Supplementary Figure 21), similar to previous studies^54^. We considered oligomers of fluorene, dibenzo[*b*,*d*]thiophene sulfone-co-phenylene, and dibenzo[*b*,*d*]thiophene sulfone as models of P1, P7, and P10 polymers, respectively. The use of oligomers as models of polymers is common practice in fully atomistic MD simulation. The strong scaling of computational cost with polymer length means that the number of atoms must be kept small enough to allow a statistically meaningful length of time to be simulated. We selected fluorene rather than phenylene (P1) for the sulfone-free material in order to eliminate the effect of differences in polymer conformation, and focus on the effect of the sulfone group. Prior studies comparing polyphenylene with a phenylene–fluorene copolymer showed that hydrogen evolution activity was similarly low in the two polymers^20^. Figure 5a, b and Supplementary Figures 22 and 23 show snapshots of hexamers of fluorene and dibenzo[*b*,*d*]thiophene sulfone in TEA–water mixtures and in reaction mixture, respectively. While the fluorene oligomer buries itself in the TEA domain, the sulfonated oligomer resides in a fine mixture of aqueous and non-aqueous domains, sometimes visible as the interface between the two domains, with its SO_2_ groups directed at the water. This preferential alignment with water tends to distort the oligomer away from its (vacuum) ground-state geometry, where the dipoles in neighboring units antialign, in order to increase contact between SO_2_ groups and water (Supplementary Figure 24). A shell of water molecules is established around the sulfone group (Supplementary Figure 25), demonstrating that the adjacency of water is a critical difference between the sulfone-free and sulfone-containing polymers. The preferential interaction with TEA for fluorene oligomers is observed equally for phenylene oligomers (Supplementary Figure 26).

Finally, we address the effect of the solvent environment on the potentials for the reactions that are associated with hydrogen evolution; primarily, the hole transfer from the polymer exciton to TEA (Fig. 4a) and the deprotonation of the TEA radical cation (Fig. 4b). These processes control the population of polarons that are available for proton reduction, which can be observed in the transient experiments in the form of the 630 nm peak. We focus on solvation in pure water and pure TEA rather than mixtures for reasons of computational tractability, but a comparison between these two limiting cases for polymers embedded in infinitely large water and TEA domains, respectively, will also give us insight into the intermediate case. To predict the potentials we use a previously reported computational approach^38,56^ based on DFT (B3LYP) calculations (see Methods) to predict the potentials of the polymers in different solvent environments, the potentials of the solution redox potentials, and the free energy of the deprotonation step (Supplementary Tables 3–5). Previously, we demonstrated that this approach performs well at reproducing the experimental potentials of conjugated polymer solids, including P1, measured in the absence of solvent by (inverse) photoemission spectroscopy^55^. The water and TEA environments are represented in these calculations using an implicit solvation model with different values for the dielectric permittivity (*ε*_r_ = 80.1 for water, *ε*_r_ = 2.38 for TEA), protons as adducts with TEA (i.e. TEAH^+^), while all polymers are modeled as oligomers containing 12 phenylene equivalent units. We only consider one to two conformers per polymer, but recent work^56^ demonstrates that the effect of the exact conformer chosen for the calculations on the predicted potentials is very small.

Figure 5c shows the potentials derived for an aqueous environment (equivalent to a pH ~11 due to the presence of TEA). The calculations predict that the polymer IP and excited state EA (EA*)—which represents the ability of an exciton to donate a hole—become steadily more positive when going from P1 to P7 to P10. Similar calculations indicate that the EA of the polymer (EA), as well as the excited state IP (IP*), steadily become less negative down the series. While the difference between IP and EA is considerably larger than the optical gap due to the relatively large exciton binding energy of conjugated polymers on the order of tenths of electronvolts, our DFT calculations reproduce the observed trend in energetic position of absorption maxima for the polymers studied here (Supplementary Figure 27). When we consider the half-reactions for the reduction of protons to hydrogen and for the *overall* oxidation of TEA to DEA and acetaldehyde (DEA (aq) + acetaldehyde (aq) + H^+^ (aq) + e^− ^-> TEA (aq)), there is a considerable (>0.5 eV) driving force for each polymer for both of these reactions in an aqueous environment. This is true for charge transfer from either the exciton or polaron state, since the calculated splitting between IP and EA*, as well as between EA and IP*, is relatively small in water. We find that the driving force for the first oxidation step, hole transfer from the polymer exciton to TEA to form the TEA radical cation (Fig. 4a), is much smaller than for the overall two-hole oxidation but that the first oxidation step is still exergonic, even for P1. A similar analysis for solvation in pure TEA (Fig. 5d) shows that all hole transfer steps become harder. Specifically, the oxidation of TEA to TEA^+^ by excitons is now endergonic for all three polymers, as is the *overall* oxidation of TEA in the case of P1. As such, *overall* oxidation of TEA by P1 excitons in a TEA environment is thermodynamically uphill, while for P7 and P10, although *overall* oxidation of TEA is still downhill, its kinetics are likely to be slowed down by the thermodynamic barrier opposing the first hole transfer step. This difference in behavior predicted for aqueous and TEA environments is due to both a larger splitting between the IP and EA* potentials of the polymers in TEA than in water, and the shift of all solution potentials to more positive values in TEA. Both effects are directly linked to the lower dielectric permittivity of TEA, which results in a weaker stabilization of charged species than in water (Supplementary Table 6). The differences in the driving force for the hole transfer step in different environments can therefore be assigned mainly to differences in the free energy of solvation.

In contrast to the hole transfer step, the driving force for deprotonation (Fig. 4b), with the proton modeled as an adduct with TEA, is always small and shows an insignificant difference in solvation energies due to the environment (Supplementary Table 7). We therefore rule out differences in the rate of deprotonation as the explanation for the lower hydrogen evolution activity of the non-polar polymer.

By combining these observations, we can now explain the difference in transient spectra and in hydrogen evolution activity for the different polymers in terms of the interactions between polymer and solvent environment. The MD simulations indicate that a non-polar polymer like P1 favors a TEA-rich environment, and the (TD)-DFT calculations predict that in the case of TEA-rich domains, hole transfer from the P1 exciton to TEA, as well as overall TEA oxidation starting from the P1 exciton, is thermodynamically uphill. By contrast, P7 and P10 favor a mixed water and TEA environment. The driving force for the different reactions should then be intermediate between those predicted for pure TEA and pure water, although likely closer to those for the water limit. In that limit transferring a hole from an exciton on P7/P10 to TEA is downhill, meaning that in contrast to the case of P1 in a TEA-rich environment there is no thermodynamic barrier to overcome, making hole transfer to TEA—and hence the overall TEA oxidation—much more favorable for these two polymers. In this way, the lack of driving force for irreversible hole transfer in the case of P1 accounts for the absence of any spectroscopic feature that could be assigned to an electron polaron in P1 and for its much lower hydrogen evolution activity. Note that this picture considers the solvent environment to be fixed on the timescale of the ps–ns transient experiments; MD simulations confirm that solvent diffusion is too slow for any significant redistribution on the ns timescale (Supplementary Figure 28).

Taken together, these experimental and computational studies suggest that multiple factors influence the efficiency of photoinduced proton reduction in the conjugated polymers in this series. Crucially, it is not possible to design polymeric photocatalysts based purely on their spectral absorption, which has been the dominant paradigm to date. Instead, it is important to consider the effect of local environment and the driving forces for individual charge transfer steps, as well as the thermodynamic potentials for overall reduction or oxidation. Importantly, polaron yields increase with the number of hydrophilic sulfone groups in the polymer backbone, and these different yields can be rationalized when taking into account the effect of the polarity of the environment on the driving force for charge transfer and particularly for TEA deprotonation. Hole transfer to TEA and TEA^•+^ deprotonation are both more strongly favored in water than in TEA, as quantified by the difference in enthalpy of solvation and thermodynamic driving forces. The sulfonated polymers attract water due to their intrinsic polarity, leading to a more polar environment where the charge and proton transfer steps will be accelerated. This suggests that surface engineering of polymer photocatalysts will be of importance in the future, as well as improving catalyst morphology to make more charges available; for example, by reducing particle size or introducing porosity. These findings are strongly relevant to the more practical goal of using polymers for overall water splitting in the absence of a SED or as a component in a Z-scheme with a second semiconductor. For example, P10 is the only catalyst here that functions well in the presence of an inorganic SED (H_2_O/Na_2_S/Na_2_SO_3_) and it also disperses much more readily in pure water than P1 or P7.

## Methods

### General methods

All reagents were obtained from Sigma-Aldrich, Fluorochem, TCI, or Strem and used as received. 3,7-Dibromodibenzo[*b*,*d*]thiophene 5,5-dioxide and 3,7-bis(4,4,5,5-tetramethyl-1,3,2-dioxaborolan-2-yl)dibenzo[*b*,*d*]thiophene 5,5-dioxide were synthesized according to a previously published procedures^57,58^. Polymer P1 and P7 were synthesized according to a previously published procedures^20^. Water for the hydrogen evolution experiments was purified using an ELGA LabWater system with a Purelab Option S filtration and ion exchange column without pH level adjustment. Reactions were carried out under nitrogen atmosphere using standard Schlenk techniques. CHN analysis was performed on a Thermo EA1112 Flash CHNS-O Analyzer using standard microanalytical procedures. Palladium content was determined via ICP-OES by Butterworth Laboratories Ltd (Teddington, UK). Transmission Fourier-transform infrared spectra were recorded on a Bruker Tensor 27 at room temperature; samples were prepared as pressed KBr pellets. Thermogravimetric analysis was performed on an EXSTAR6000 by heating samples at 10 °Cmin^−1^ under air in open aluminum pans from 40 to 600 °C and holding at 600 °C for 30 min. The UV–Vis absorption spectra of the polymers in the solid state were recorded on a Shimadzu UV-2550 UV–Vis spectrometer, in the reflectance spectroscopy geometry with a diffuse reflectivity (integrating sphere) attachment. The photoluminescence spectra of the polymer were measured with a Shimadzu RF-5301PC fluorescence spectrometer. UV–Vis absorption spectra of the polymers dispersed in waters (0.1 mg mL^−1^) were recorded using a dual-beam Shimadzu UV-2600 spectrophotometer fitted with an integrating sphere attachment, in order to maximally suppress reflectance and scattering losses. The same consideration motivated the use of the thin, 1 mm light path Suprasil quartz cuvettes (Hellma 110-QS, 350 μL) for carrying out the measurements in aqueous suspensions. PL spectra of such suspensions were recorded in standard reflection geometry using a Horiba FluoroMax-3 spectrofluorometer with excitations at 345 nm for P1 polymer, and 360 nm for P7 and P10 polymers. All powder- and suspension-based UV–Vis absorption and photoluminescence measurements were performed at room temperature. Imaging of the polymer morphology was achieved on a Hitachi S4800 Cold Field Emission SEM, with secondary electron, backscatter, and transmission detectors. PXRD data were collected in high-throughput transmission mode on a Panalytical Empyrean diffractometer producing Cu-Ka (*λ* = 1.5418 Å) radiation, equipped with an X-ray focusing mirror and PIXcel 3D detector. Static contact angle measurements with the sessile drop method were recorded and analyzed at room temperature on a Krüss DSA100 instrument measured in at least three different locations with water and water/methanol (1:1 mixtures). Nitrogen sorption isotherms were measured using Micromeritics 2420 volumetric adsorption analyzer. Surface areas were calculated in the relative pressure (*P*/*P*_0_) range from 0.01 to 0.10 of the adsorption branch.

### Solid-state NMR experiments

The solid-state NMR experiment was performed on a 400 MHz 9.4 T Bruker Avance III HD solid-state NMR spectrometer equipped with a 4 mm HXY triple-resonance magic angle spinning (MAS) probe (in double resonance mode) with the ^1^H channel tuned to ^1^H at *ν*_0_(1H) = 400.13 MHz and the X channel tuned to ^13^C at *ν*_0_(^13^C) = 100.61 MHz. The experiment was performed at room temperature under MAS at 12.5 kHz. ^1^H pulses and SPINAL-64 heteronuclear decoupling^59^ performed at a radio-frequency (rf) field amplitude of 83 kHz. ^13^C cross-polarization (CP) MAS experiments were obtained with a ^13^C rf field of 60 kHz, while the ^1^H rf field amplitude was ramped to obtain maximum signal at a ^1^H rf field of approximately 60 kHz, and at a contact time of 2 ms. Three thousand seventy-two scans were accumulated with a 3 s recycle delay. The ^13^C chemical shifts were referenced to the CH carbon of adamantane at 29.45 ppm^60^. Samples were packed in a zirconia rotor with a KelF cap and NMR data were obtained with TopSpin 3.2 and analyzed using TopSpin 3.5^59^. ^13^C MAS NMR spectrum assignment was performed by identifying the protonated carbons by recording the NMR spectrum with a short contact time of 0.1 ms, and using data from the literature^20^ and chemical shift databases^61^. The shoulder at 121 ppm is tentatively assigned to the ***C***-Br of starting material or end-group on the polymer chain^62^. The shoulder at 121 ppm is tentatively assigned to the ***C***-Br of starting material or end-group on the polymer chain.

The ^13^C solid-state NMR spectrum (Supplementary Figure 1) showed a range of relatively well-resolved resonances in the 100 to 150 ppm region that could be assigned to all anticipated carbons; for example, the -SCCH***C***-carbon appears at 130 ppm rather than ~120 ppm in 2-bromo[*b*,*d*]dibenzothiophene^62^ which confirms polymerization. Note that the latter signal is still present in P10 showing that either low level of the dibromoarenes exist or that there is a significant population of brominated end groups.

### Synthesis of P10 via Suzuki–Miyaura-type polycondensation^20^

A flask was charged with the 3,7-dibromodibenzo[*b*,*d*]thiophene 5,5-dioxide (281 mg, 0.75 mmol), 3,7-bis(4,4,5,5-tetramethyl-1,3,2-dioxaborolan-2-yl)dibenzo[*b*,*d*]thiophene 5,5-dioxide (351 mg, 0.75 mmol), *N*,*N*-dimethylformamide (20 mL), an aqueous solution of K_2_CO_3_ (4 mL, 2.0M), and [Pd(PPh_3_)_4_] (15 mg). The mixture was degassed by bubbling with N_2_ for 30 min and heated to 150 °C for 2 days. The mixture was cooled to room temperature and poured into water. The precipitate was collected by filtration and washed with H_2_O and methanol. Further purification of the polymers was carried out by Soxhlet extraction with chloroform to remove any low-molecular weight by-products. The product was dried under reduced pressure and obtained as a yellow powder (290 mg, 90%). Anal. calcd for (C_12_H_6_O_2_S)_*n*_: C, 67.28; H, 2.82; S, 14.96%; Found C, 60.27; H, 2.87; S, 13.87%. Note: The yields were calculated ignoring the presence of end functional groups whose nature is unclear.

### Synthesis of P10Y via oxidative Yamamoto coupling

A flame-dried Schlenk flask was charged with 3,7-dibromodibenzo[*b*,*d*]thiophene 5,5-dioxide (374 mg, 1.00 mmol), 2,2′-bipyridine (344 mg, 2.20 mmol), and transferred into a dry glove-box. Inside the glove-box the flask was charged with bis(cyclooctadiene)nickel(0) (660 mg, 2.40 mmol). Outside the glove-box 1,5-cyclooctadiene (338 mg, 2.20 mmol) and *N*,*N*-dimethylformamide (anhydrous, 20 mL) were added and the resulting suspension was heated to 80 °C under nitrogen for 2 days. After cooling to room temperature hydrochloric acid was added (conc., 20 mL) and the polymer was filtered off. The polymer was washed with water until neutral, and then methanol and tetrahydrofuran. Further purification of the polymers was carried out by Soxhlet extraction with chloroform to remove any low-molecular weight by-products and the product was dried under reduced pressure. The product was obtained as a yellow powder (192 mg, 90%). Anal. calcd for (C_12_H_6_O_2_S)_*n*_: C, 67.28; H, 2.82; S, 14.96%; Found C, 60.55; H, 3.87; S, 11.23%. Note: The yields were calculated ignoring the presence of end functional groups whose nature is unclear.

### Hydrogen evolution experiments

A flask was charged with the polymer powder (25 mg), water, triethylamine, methanol (1:1:1 volume mixture, 25 mL), and sealed with a septum. The resulting suspension was ultrasonicated until the photocatalyst was dispersed before degassing by N_2_ bubbling for 30 min. The reaction mixture was illuminated with a 300 W Newport Xe light source (Model: 6258, Ozone free) for the time specified under atmospheric pressure. The Xe light source was cooled by water circulating through a metal jacket. An Oriel Instruments LSH-7320 (IEC ABA certified) Solar Simulator under 1 Sun illumination was used for the samples specified aligned using the instrument laser diode. Gas samples were taken with a gas-tight syringe and run on a Bruker 450-GC gas chromatograph equipped with a Molecular Sieve 13X 60–80 mesh 1.5 m × 1/8 in. × 2 mm ss column at 50 °C with an argon flow of 40.0 mL min^−1^. Hydrogen was detected with a thermal conductivity detector referencing against standard gas with a known concentration of hydrogen. Hydrogen dissolved in the reaction mixture was not measured and the pressure increase generated by the evolved hydrogen was neglected in the calculations. The rates were determined from a linear regression fit and the error is given as the standard deviation of the amount of hydrogen evolved. No hydrogen evolution was observed for a mixture of water/methanol/trimethylamine under *λ* > 295 nm illumination in absence of a photocatalyst. Filter for the wavelength dependency experiments were obtained from Edmund Optics Ltd (UK). Transmission spectra of the filters and output profiles of the light sources are shown in Supplementary Figure 29.

The external quantum efficiency for the photocatalytic H_2_ evolution was measured using a *λ * = 420 nm LED controlled by an IsoTech IPS303DD power supply. For the experiments, the polymer (12 mg) was suspended in water, triethylamine, and methanol (1:1:1 volume mixture). An area of 8 cm^2^ was illuminated and the light intensity was measured with a ThorLabs S120VC photodiode power sensor controlled by a ThorLabs PM100D Power and Energy Meter Console. The external quantum efficiencies were estimated using the equation below:

$${\mathrm{EQE\% }} = 2 \times \frac{{\mathrm{moles}}\hskip 2pt {\mathrm{of}}\hskip 2pt {\mathrm{hydrogen}}\hskip 2pt {\mathrm{evolved}}}{{\mathrm{moles}}\hskip 2pt {\mathrm{of}}\hskip 2pt {\mathrm{incident}}\hskip 2pt {\mathrm{photons}}} \times 100\%.$$

### Transient spectroscopy sample preparation

Dispersions of P1, P7, and P10 with a concentration of 1.67 g L^−1^ were prepared in water and ultrasonicated to enhance dispersability. TAS samples with a concentration of 0.24 g L^−1^ were then prepared from this dispersion using the given solvent ratios in quartz cuvettes with a path length of 2 mm (Hellma Suprasil quartz 700 μL). Cuvettes were sealed using rubber septa caps and carefully purged with argon for 20 min. Dispersions were stirred using a magnetic stirrer during the ultrafast TAS measurements to prevent settling due to the longer single measurements as compared to those on the μs–s timescale.

### Transient absorption spectroscopy (fs–ns)

Our ultrafast transient absorption setup has been described in detail elsewhere^63^. In addition to the near infrared wavelength continuum, a wavelength continuum in the visible range was generated for the present study by focusing the 800 nm amplifier output into a Ti:sapphire crystal. Spectra were corrected for group velocity dispersion using the software Surface Xplorer 4.0 (Ultrafast Systems). The acquired data was processed in OriginPro 2015/2017.

### Transient absorption spectroscopy (µs–s)

Transient absorption data on the µs–s timescale was acquired using a home-built transient absorption spectrometer, where the third harmonic output of a Nd:YAG laser (OPOTEK Opolette 355 II, 4–7 ns pulse width) was used for 355 nm excitation. The laser output is transmitted to the sample via a liquid light guide. Samples were excited at an excitation fluence of 0.32 mJ cm^−2^ and excitation fluences were measured using a pyroelectric energy sensor (Ophir Photonics PE9). The monochromated output of a 100 W quartz halogen lamp (Bentham IL1) is used as a probe beam and is recorded by a Si photodiode detector (Hamamatsu S3071) after passing through the sample. Appropriate long-pass filters were positioned between sample and detector to attenuate scattered laser light. Data acquisitions are triggered by a photodiode (Thorlabs DET210) using scattered laser light. Data were recorded in a home-built LabVIEW-based software with an oscilloscope (Tektronix DPO 2012B) after amplification on the µs–ms timescale (Costronics 1999 amplifier) and simultaneously with a DAQ card (National Instruments, NI USB-6211) on the ms–s timescale. The kinetic traces shown were typically obtained as an average of 40 individual excitation events with the subtraction of laser scatter. The acquired data was processed in OriginPro 2015/2017.

### Dynamic light scattering

The viscosity of reaction mixture was measured with a Brookfield LVDV-I Prime digital viscometer. A Brookfield LV-2C cylindrical spindle was driven at fixed rotational speeds of 30, 50, 60, and 100 rpm while immersed in 21 mL of reaction mixture. The solvent temperature was held at 25 °C using a heat jacket surrounding the cylinder, with water provided by a Grant W6 waterbath. The dynamic viscosity of the water/methanol/triethylamine (equal volumes) mixture was determined as 2.36 ± 0.0 cP at all four rotation speeds, indicating that reaction mixture is a Newtonian fluid. The dynamic viscosity of reaction mixture in DLS experiments—where the shear rate is zero—was therefore taken to be 2.36 cP.

Water-based samples were measured in disposable polystyrene cuvettes, while reaction mixture-based suspensions were measured in quartz cuvettes. Cuvettes were first thoroughly cleaned with water and isopropanol (IPA) before being rinsed with the dispersant solvent; quartz cuvettes were also cleaned with acetone first. All dispersants were twice filtered using 200 nm polytetrafluoroethylene filters to minimize dust contamination. The powdered polymers were dispersed in the solvent of choice at a concentration of 0.25 mg mL^−1^, sonicated for 1 h for maximal homogeneity, and then transferred to the cuvettes. Cuvette surfaces were wiped with an IPA lint-free cloth immediately before measurement.

DLS measurements were performed using a Malvern Zetasizer Nano S with scattered light collected at 173° to the incoming 633 nm laser beam. All samples were held at 25 °C during measurement. Three measurements of 5 min were recorded for each sample, with two samples measured per polymer–solvent system. Typical intensity correlation functions are shown in Supplementary Figure 30. Further methodological details can be found under Supplementary Methods.

### (TD-)DFT calculations

Dipole moments of the different polymer building blocks were calculated using the B3LYP density functional^64,65^ and the 6–311g(d,p)basis-set^66,67^ (see Supplementary Methods for further details).

The potentials of oligomeric models of the polymers in different solvent environments, the potentials of the solution redox potentials, and the free energy of the deprotonation step were obtained from (TD-)DFT calculations using the B3LYP density functional^64,65^, the DZP basis-set^68^ and the COSMO solvation model^69^. Dielectric permittivity values of 80.1 for water and 2.38 for TEA were used and protons were represented as adducts with TEA (TEAH^+^). In the case of solution species, the Gibbs free energy includes contributions from the internal energy, solvation free energy, as well as those arising from the vibrational, translational, and rotational degrees of freedom. For the polymer models, the latter contributions were neglected as they were previously shown to be small^38^. The calculated potential values are converted from the vacuum scale to that of the standard hydrogen electrode (SHE) by shifting them by the experimentally obtained value of the SHE absolute potential (SHEAP). Here we use, in line with our previous work, the International Union of Pure and Applied Chemistry-proposed value for the SHEAP of 4.44 V^70^. See Supplementary Methods for more details about the used methodology. The conformers considered for each of the oligiomeric models of the polymers correspond to low-energy conformations obtained through a conformer search using the Schrodinger PLC Macromodel software and the OPLS2005^71,72^ forcefield. The (TD-)DFT total energies and *xyz* coordinates of the polymer models in all relevant (charge) states, finally, can be found Supplementary Data 1 and Supplementary Data 2–15, respectively.

### MD simulations

MD simulations were carried out using the GROMACS 5.1.4 package^73^ and force fields are based on OPLS-AA^71^. Initial configurations were made using Gromacs and PACKMOL^74^. All simulations snapshots shown were from NPT simulations with a timestep of 2 fs and using the Berendsen thermostat and barostats. The procedure used is shown in Supplementary Methods, while the forcefield details are outlined in Supplementary Figure 31 as well as Supplementary Tables 8–10.

## Electronic supplementary material

Supporting Information file

Description of Additional Supplementary Files

Supplementary Data 1

Supplementary Data 2

Supplementary Data 3

Supplementary Data 4

Supplementary Data 5

Supplementary Data 6

Supplementary Data 7

Supplementary Data 8

Supplementary Data 9

Supplementary Data 10

Supplementary Data 11

Supplementary Data 12

Supplementary Data 13

Supplementary Data 14

Supplementary Data 15

## Data Availability

All data generated or analyzed in this study are included in article and the supplementary files.

## References

[1] Ong WJJ, Tan LLL, Ng YH, Yong STT, Chai SPP (2016). Graphitic carbon nitride (g-C_3_N_4_)-based photocatalysts for artificial photosynthesis and environmental remediation: are we a step closer to achieving sustainability?. Chem. Rev..

[2] Vyas VS, Lau VWH, Lotsch BV (2016). Soft photocatalysis: organic polymers for solar fuel production. Chem. Mater..

[3] Zhang G, Lan ZA, Wang X (2016). Conjugated polymers: catalysts for photocatalytic hydrogen evolution. Angew. Chem. Int. Ed..

[4] Kudo A, Miseki Y (2009). Heterogeneous photocatalyst materials for water splitting. Chem. Soc. Rev..

[5] Li Y, Xu H, Ouyang S, Ye J (2016). Metal–organic frameworks for photocatalysis. Phys. Chem. Chem. Phys..

[6] Wang X (2009). A metal-free polymeric photocatalyst for hydrogen production from water under visible light. Nat. Mater..

[7] Sun J (2012). Bioinspired hollow semiconductor nanospheres as photosynthetic nanoparticles. Nat. Commun..

[8] Stegbauer L, Schwinghammer K, Lotsch BV (2014). A hydrazone-based covalent organic framework for photocatalytic hydrogen production. Chem. Sci..

[9] Lau VW (2016). Rational design of carbon nitride photocatalysts by identification of cyanamide defects as catalytically relevant sites. Nat. Commun..

[10] Zhang G (2017). Optimizing optical absorption, exciton dissociation, and charge transfer of a polymeric carbon nitride with ultrahigh solar hydrogen production activity. Angew. Chem. Int. Ed..

[11] Martin DJ, Reardon PJT, Moniz SJA, Tang J (2014). Visible light-driven pure water splitting by a nature-inspired organic semiconductor-based system. J. Am. Chem. Soc..

[12] Zhang G, Lan ZA, Lin L, Lin S, Wang X (2016). Overall water splitting by Pt/g-C_3_N_4_ photocatalysts without using sacrificial agents. Chem. Sci..

[13] Pan Z, Zheng Y, Guo F, Niu P, Wang X (2017). Decorating CoP and Pt nanoparticles on graphitic carbon nitride nanosheets to promote overall water splitting by conjugated polymers. ChemSusChem.

[14] Yanagida S, Kabumoto A, Mizumoto K, Pac C, Yoshino K (1985). Poly(*p*-phenylene)-catalysed photoreduction of water to hydrogen. J. Chem. Soc. Chem. Commun..

[15] Shibata T (1990). Novel visible-light-driven photocatalyst. Poly(*p*-phenylene)-catalyzed photoreductions of water, carbonyl compounds, and olefins. J. Phys. Chem..

[16] Matsuoka S, Kohzuki T, Kuwana Y, Nakamura A, Yanagida S (1992). Visible-light-induced photocatalysis of poly(pyridine-2,5-diyl). Photoreduction of water, carbonyl compounds and alkenes with triethylamine. J. Chem. Soc. Perkin Trans..

[17] Yanagida, S. et al. Synthesis of 2,′:5′,2″-terpyridine and 2,2′:5′,2″:5″,2‴-quaterpyridine and their photocatalysis of the reduction of water. *J. Chem. Soc.* *Perkin Trans*. **2**, 1963–1969 (1996).

[18] Bi J (2015). Covalent triazine-based frameworks as visible light photocatalysts for the splitting of water. Macromol. Rapid Commun..

[19] Bornoz P, Prévot MS, Yu X, Guijarro N, Sivula K (2015). Direct light-driven water oxidation by a ladder-type conjugated polymer photoanode. J. Am. Chem. Soc..

[20] Sprick RS (2016). Visible-light-driven hydrogen evolution using planarized conjugated polymer photocatalysts. Angew. Chem. Int. Ed..

[21] Yang C (2016). Molecular engineering of conjugated polybenzothiadiazoles for enhanced hydrogen production by photosynthesis. Angew. Chem. Int. Ed..

[22] Wang L (2016). Organic polymer dots as photocatalysts for visible light-driven hydrogen generation. Angew. Chem. Int. Ed..

[23] Sprick RS (2015). Tunable organic photocatalysts for visible-light-driven hydrogen evolution. J. Am. Chem. Soc..

[24] Sprick RS (2016). Extended conjugated microporous polymers for photocatalytic hydrogen evolution from water. Chem. Commun..

[25] Li L, Lo WY, Cai Z, Zhang N, Yu L (2016). Donor–acceptor porous conjugated polymers for photocatalytic hydrogen production: The importance of acceptor comonomer. Macromolecules.

[26] Wang L (2017). Conjugated Microporous polymer nanosheets for overall water splitting using visible light. Adv. Mater..

[27] Li L (2016). Rational design of porous conjugated polymers and roles of residual palladium for photocatalytic hydrogen production. J. Am. Chem. Soc..

[28] Vyas VS (2015). A tunable azine covalent organic framework platform for visible light-induced hydrogen generation. Nat. Commun..

[29] Schwab MG (2010). Photocatalytic hydrogen evolution through fully conjugated poly(azomethine) networks. Chem. Commun..

[30] Woods DJ, Sprick RS, Smith CL, Cowan AJ, Cooper AI (2017). A solution-processable polymer photocatalyst for hydrogen evolution from water. Adv. Energy Mater..

[31] Li L (2016). Photocatalysts based on cobalt-chelating conjugated polymers for hydrogen evolution from water. Chem. Mater..

[32] Pati PB (2017). An experimental and theoretical study of an efficient polymer nano-photocatalyst for hydrogen evolution. Energy Environ. Sci..

[33] Tseng PJ (2018). Design and synthesis of cycloplatinated polymer dots as photocatalysts for visible light-driven hydrogen evolution. ACS Catal..

[34] Vyas VS, Lotsch BV (2015). Materials chemistry: organic polymers form fuel from water. Nature.

[35] Sprick RS (2018). Nitrogen containing linear poly(phenylene) derivatives for photo-catalytic hydrogen evolution from water. Chem. Mater..

[36] Sprick RS (2018). Maximising the hydrogen evolution activity in organic photocatalysts by co-polymerisation. J. Mater. Chem. A.

[37] Clarke TM, Durrant JR (2010). Charge photogeneration in organic solar cells. Chem. Rev..

[38] Guiglion P, Butchosa C, Zwijnenburg MA (2016). Polymer photocatalysts for water splitting: insights from computational modeling. Macromol. Chem. Phys..

[39] Maeda K (2009). Photocatalytic activities of graphitic carbon nitride powder for water reduction and oxidation under visible light. J. Phys. Chem. C.

[40] Meier CB (2017). Structure-property relationships for covalent triazine-based frameworks: the effect of spacer length on photocatalytic hydrogen evolution from water. Polymer.

[41] Xu S, Klimov VI, Kraabel B, Wang H, McBranch DW (2001). Femtosecond transient absorption study of oriented poly(9,9-dioctylfluorene) film: hot carriers, excitons, and charged polarons. Phys. Rev. B.

[42] Kraabel B (2000). Unified picture of the photoexcitations in phenylene-based conjugated polymers: universal spectral and dynamical features in subpicosecond transient absorption. Phys. Rev. B.

[43] Stevens MA, Silva C, Russell DM, Friend RH (2001). Exciton dissociation mechanisms in the polymeric semiconductors poly(9,9-dioctylfluorene) and poly(9,9-dioctylfluorene-co-benzothiadiazole). Phys. Rev. B.

[44] Frankevich E (2000). Formation of polaron pairs and time-resolved photogeneration of free charge carriers in π-conjugated polymers. Phys. Rev. B.

[45] Müller JG, Lemmer U, Feldmann J, Scherf U (2002). Precursor states for charge carrier generation in conjugated polymers probed by ultrafast spectroscopy. Phys. Rev. Lett..

[46] Pesci FM, Cowan AJ, Alexander BD, Durrant JR, Klug DR (2011). Charge carrier dynamics on mesoporous WO_3_ during water splitting. J. Phys. Chem. Lett..

[47] Wang X (2015). Transient absorption spectroscopy of anatase and rutile: the Impact of morphology and phase on photocatalytic activity. J. Phys. Chem. C.

[48] Reynal A, Lakadamyali F, Gross MA, Reisner E, Durrant JR (2013). Parameters affecting electron transfer dynamics from semiconductors to molecular catalysts for the photochemical reduction of protons. Energy Environ. Sci..

[49] Rodenberg A (2015). Mechanism of photocatalytic hydrogen generation by a polypyridyl-based cobalt catalyst in aqueous solution. Inorg. Chem..

[50] Pastor E (2017). Spectroelectrochemical analysis of the mechanism of (photo)electrochemical hydrogen evolution at a catalytic interface. Nat. Commun..

[51] Kroeze JE, Savenije TJ, Vermeulen MJW, Warman JM (2003). Contactless determination of the photoconductivity action spectrum, exciton diffusion length, and charge separation efficiency in polythiophene-sensitized TiO_2_ bilayers. J. Phys. Chem. B.

[52] Bruno A, Reynolds LX, Dyer-Smith C, Nelson J, Haque SA (2013). Determining the exciton diffusion length in a polyfluorene from ultrafast fluorescence measurements of polymer/fullerene blend films. J. Phys. Chem. C.

[53] Shaw PE, Ruseckas A, Samuel IDW (2008). Exciton diffusion measurements in poly(3-hexylthiophene). Adv. Mater..

[54] Kajimoto S, Yoshii N, Hobley J, Fukumura H, Okazaki S (2007). Electrostatic potential gap at the interface between triethylamine and water phases studied by molecular dynamics simulation. Chem. Phys. Lett..

[55] Wilbraham, L., Berardo, E., Turcani, L., Jelfs, K. & Zwijnenburg, M. A high-throughput screening approach for the optoelectronic properties of conjugated polymers. *J. Chem. Inf. Model*10.1021/acs.jcim.8b00256 (2018). 10.1021/acs.jcim.8b00256 PMC6307085 29940733

[56] Guiglion P, Monti A, Zwijnenburg MA (2017). Validating a density functional theory approach for predicting the redox potentials associated with charge carriers and excitons in polymeric photocatalysts. J. Phys. Chem. C.

[57] Sprick RS (2013). Triarylamine polymers of bridged phenylenes by (*N*-heterocyclic carbene)-palladium catalysed C–N coupling. J. Mater. Chem. C.

[58] Moss KC (2010). Tuning the intramolecular charge transfer emission from deep blue to green in ambipolar systems based on dibenzothiophene S, S-dioxide by manipulation of conjugation and strength of the electron donor units. J. Org. Chem..

[59] Fung BM, Khitrin AK, Ermolaev K (2000). An improved broadband decoupling sequence for liquid crystals and solids. J. Magn. Reson..

[60] Morcombe CR, Zilm KW (2003). Chemical shift referencing in MAS solid state NMR. J. Magn. Reson..

[61] Silverstein, R. M., Webster, F. X., Kiemle, D. J. & Bryce, D. L. *Spectrometric Identification of Organic Compounds* 8th edn (Wiley, United States, 2004).

[62] Jayalakshmi S, Perumal S, Wilson DA (1989). Proton and carbon NMR spectra of 2-substituted dibenzothiophenes. Magn. Reson. Chem..

[63] Sachs M, Pastor E, Kafizas A, Durrant JR (2016). Evaluation of surface state mediated charge recombination in anatase and Rutile TiO_2_. J. Phys. Chem. Lett..

[64] Becke AD (1993). Density‐functional thermochemistry. III. The role of exact exchange. J. Chem. Phys..

[65] Stephens PJ, Devlin FJ, Chabalowski CF, Frisch MJ (1994). *Ab initio* calculation of vibrational absorption and circular dichroism spectra using density functional force fields. J. Phys. Chem..

[66] Krishnan R, Binkley JS, Seeger R, Pople JA (1980). Self‐consistent molecular orbital methods. XX. A basis set for correlated wave functions. J. Chem. Phys..

[67] McLean AD, Chandler GS (1980). Contracted Gaussian basis sets for molecular calculations. I. Second row atoms, *Z* =11–18. J. Chem. Phys..

[68] Schäfer A, Horn H, Ahlrichs R (1992). Fully optimized contracted Gaussian basis sets for atoms Li to Kr. J. Chem. Phys..

[69] Klamt A, Schüürmann G (1993). COSMO: a new approach to dielectric screening in solvents with explicit expressions for the screening energy and its gradient. J. Chem. Soc. Perkin Trans..

[70] Trassati, S. The absolute electrode potential: an explanatory note. *Pure & Appl. Chem.*, **58**, 955–966 (1986).

[71] Jorgensen WL, Maxwell DS, Tirado-Rives J (1996). Development and testing of the OPLS all-atom force field on conformational energetics and properties of organic liquids. J. Am. Chem. Soc..

[72] Banks JL (2005). Integrated modeling program, applied chemical theory (IMPACT). J. Comput. Chem..

[73] Berendsen HJC, van der Spoel D, van Drunen R (1995). GROMACS: a message-passing parallel molecular dynamics implementation. Comput. Phys. Commun..

[74] Martínez L, Andrade R, Birgin EG, Martínez JM (2009). PACKMOL: a package for building initial configurations for molecular dynamics simulations. J. Comput. Chem..

