# Supplementary material for: Understanding structure-activity relationships in linear polymer photocatalysts for hydrogen evolution
Source: Nat Commun. 2018 Nov 23;9:4968. doi: 10.1038/s41467-018-07420-6 (PMC6251929; doi:10.1038/s41467-018-07420-6)
Supplement: Supplementary file 1 — Supporting Information file [file 41467_2018_7420_MOESM1_ESM.pdf]

Understanding structure-activity relationships in linear polymer photocatalysts for hydrogen evolution

Sachs, Sprick, et. al.

Supplementary Figures

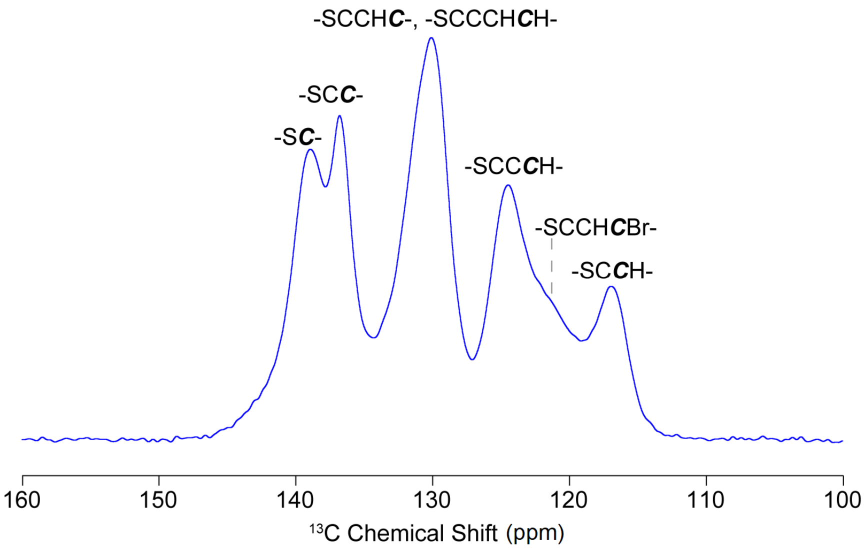

Supplementary Figure 1. ^{13}C CP MAS NMR of P10 with full assignment, including a minor resonance ascribed to *C*-Br, either from residual starting material or polymeric end groups.

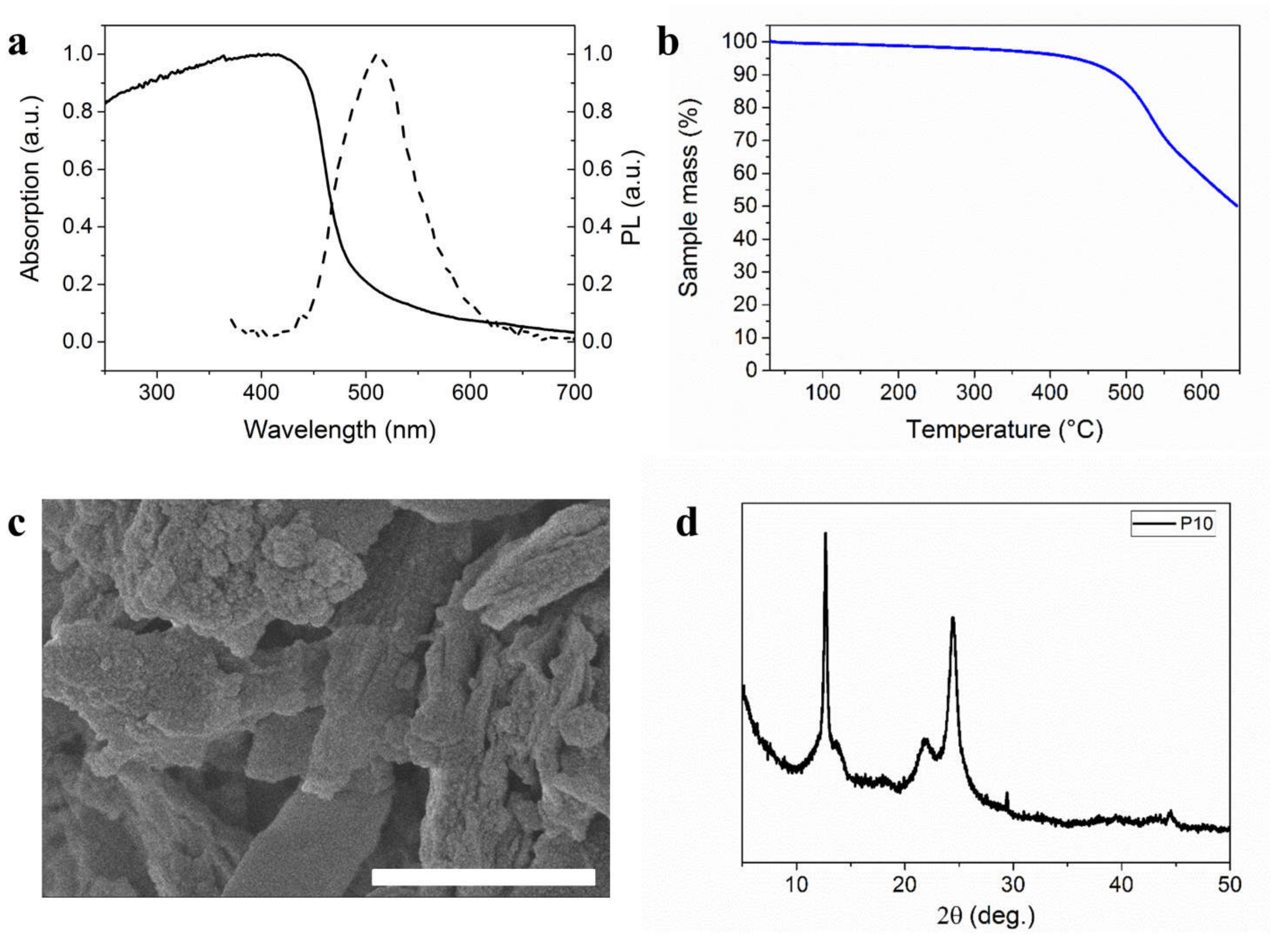

Supplementary Figure 2. a Solid-state UV-vis and photoluminescence (PL) spectrum of P10 **b** thermogravimetric analysis (TGA) of P10 heated at 10 °C min^{-1} under nitrogen **c** scanning electron microscopy image of P10 (the white bar is 1 µm long) **d** powder X-ray diffraction pattern of P10.

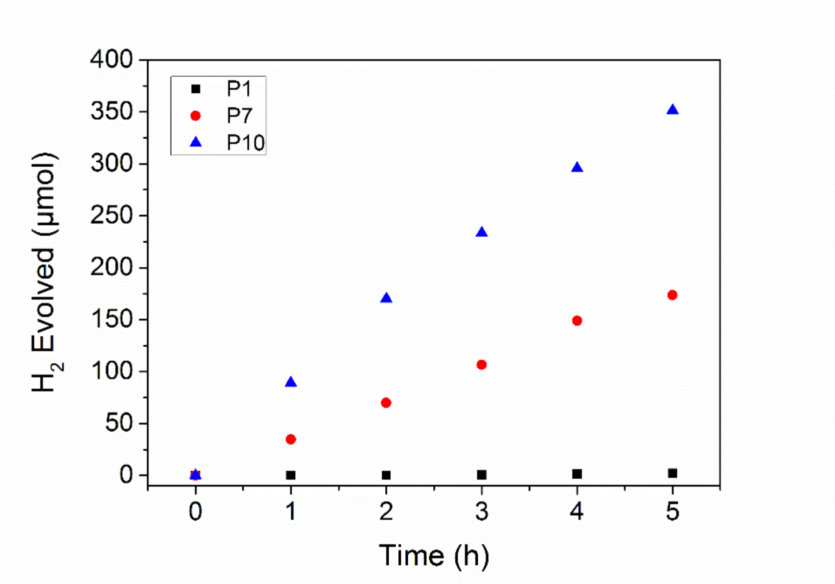

Supplementary Figure 3. Hydrogen evolution of P1, P7, and P10 (25 mg) from water / triethylamine (5 vol. %) under λ > 420 nm irradiation.

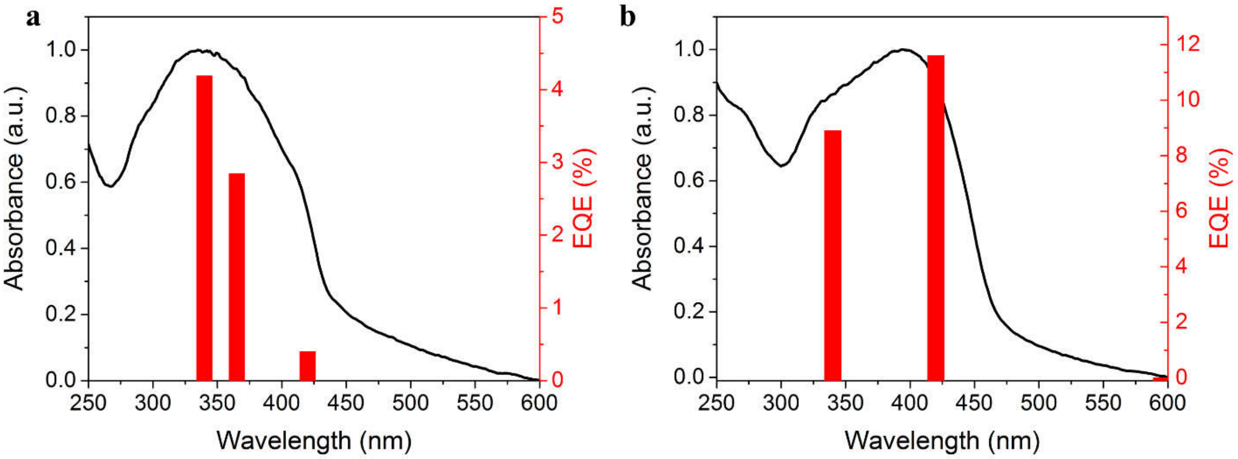

Supplementary Figure 4. External quantum efficiencies of **a** P1 (12 mg) from a triethylamine/water/methanol mixture (8 mL) at 340, 365, and 420 nm (± 10 nm, fwhm LEDs), **b** P10 (12 mg) from a triethylamine/water/methanol mixture (8 mL) at 340, 420, and 595 nm (± 10 nm, fwhm LEDs).

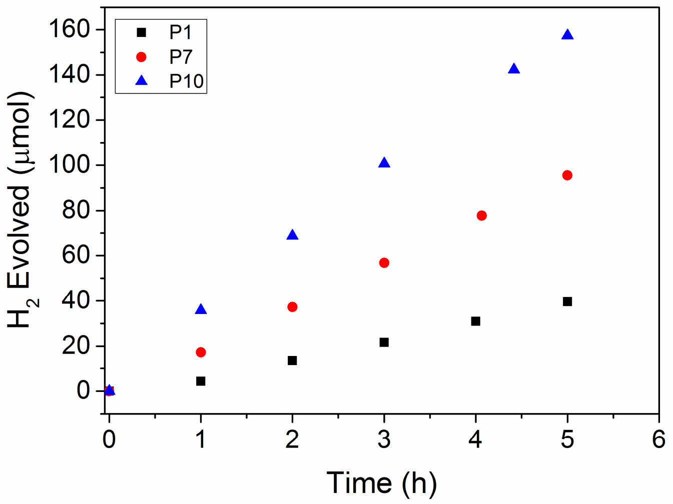

Supplementary Figure 5. Hydrogen evolution of P1, P7, and P10 (25 mg) from water/methanol/TEA under UV irradiation using a U-340 filter (transmissive in the range of 255 to 395 nm).

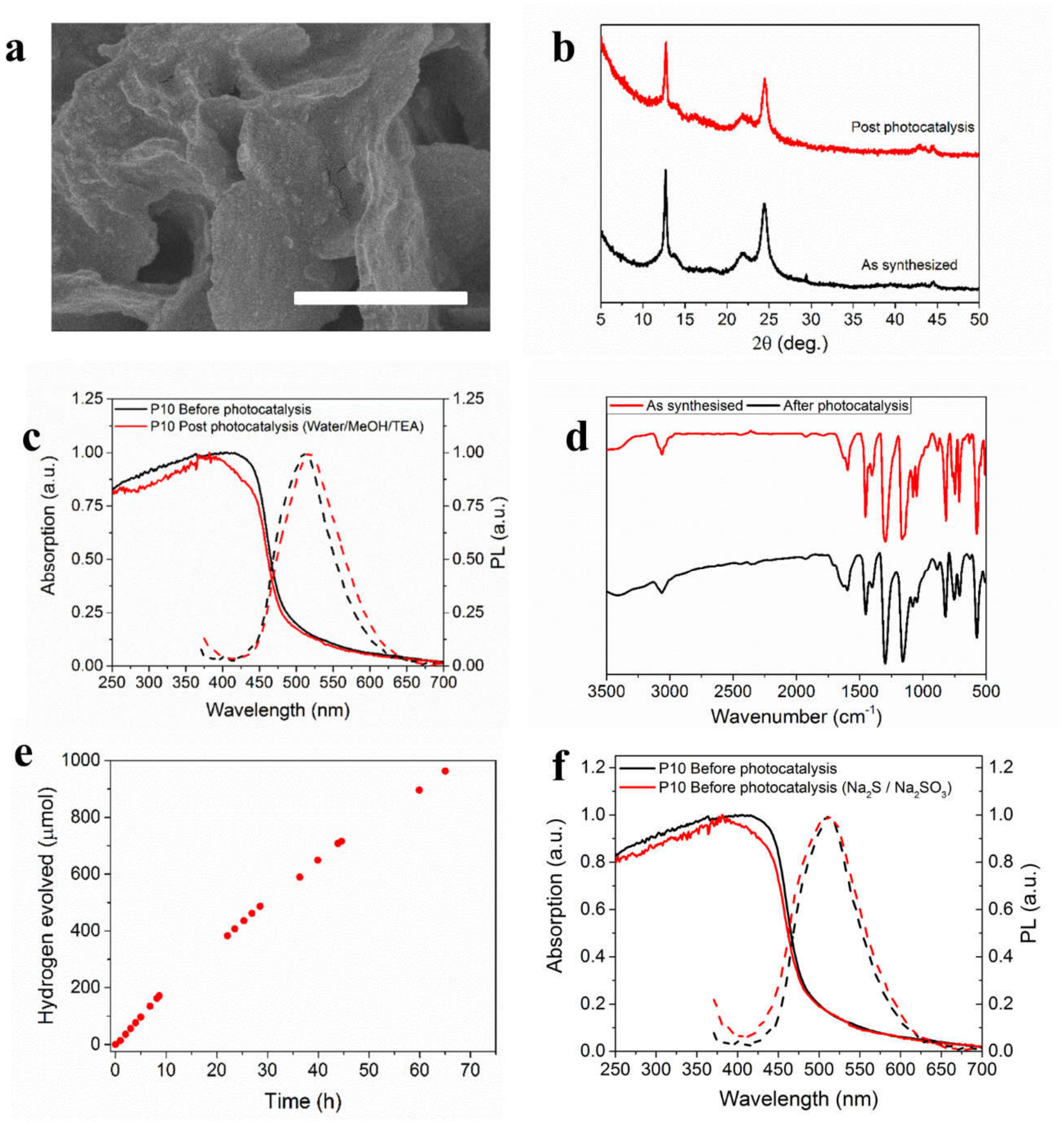

Supplementary Figure 6. Analysis of P10 after 40 hours of a hydrogen evolution of experiment from a triethylamine/water/methanol mixture under λ > 420 nm illumination **a** scanning electron microscopy image (the white bar is 1 µm long) **b** powder X-ray diffraction pattern before and after photolysis **c** UV/Vis and photoluminescence spectra measured in the solid-state before and after photolysis **d** Fourier transform infrared spectra before and after photolysis **e** Hydrogen evolution of P10 (25 mg) from water / Na_2S (0.35 M) / Na_2SO_3 (0.25 M) under λ > 420 nm irradiation for more than 60 hours **f** UV/Vis and photoluminescence spectra before and after photolysis measured in the solid-state.

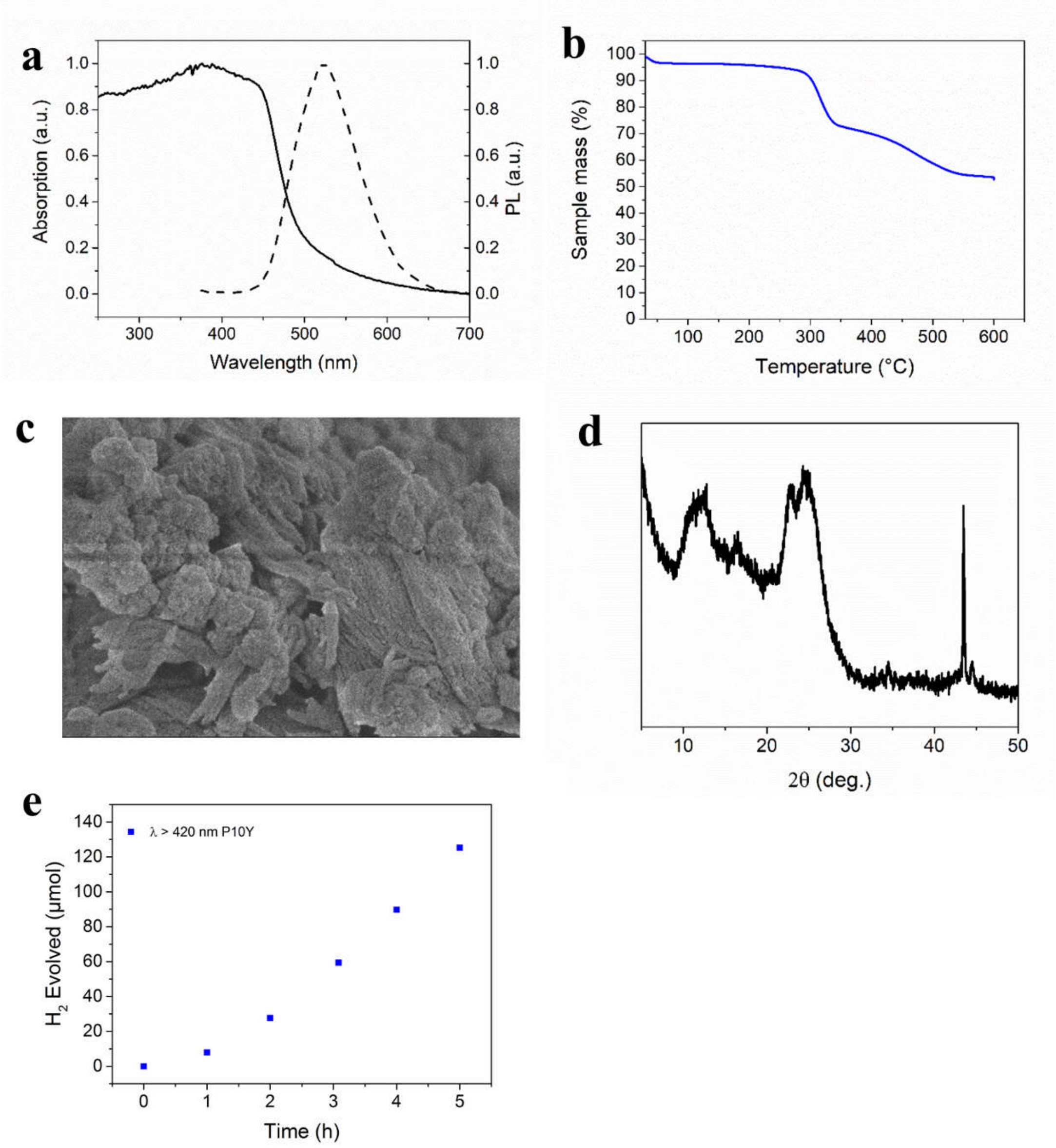

Supplementary Figure 7. a Solid-state UV-vis and photoluminescence (PL) spectrum of P10Y **b** thermogravimetric analysis (TGA) of P10Y heated at 10 °C min^{-1} under nitrogen **c** scanning electron microscopy image of P10Y (the white bar is 1 μm long) **d** powder X-ray diffraction pattern of P10Y **e** photocatalytic hydrogen production of P10Y (25 mg) from water/methanol/triethylamine (1:1:1 mixture) under λ > 420 nm irradiation.

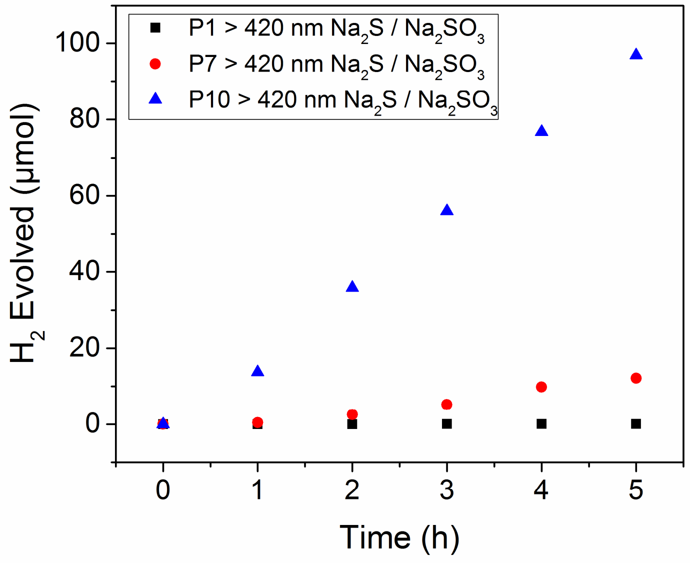

Supplementary Figure 8. Hydrogen evolution of P1, P7, and P10 (25 mg) from water / Na_2S (0.35 M) / Na_2SO_3 (0.25 M) under λ > 420 nm Xe-lamp (300 W) irradiation.

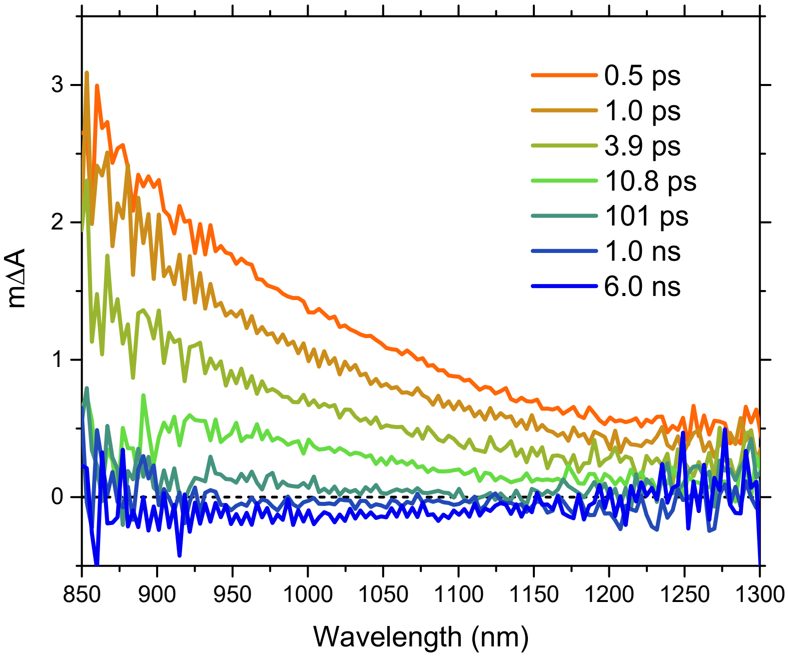

Supplementary Figure 9. Transient absorption spectrum obtained from a P10 suspension in a solvent mixture consisting of equal volumes of H_2O, MeOH, and TEA using an excitation wavelength of 355 nm and a fluence of 0.08 mJ cm^{-2}.

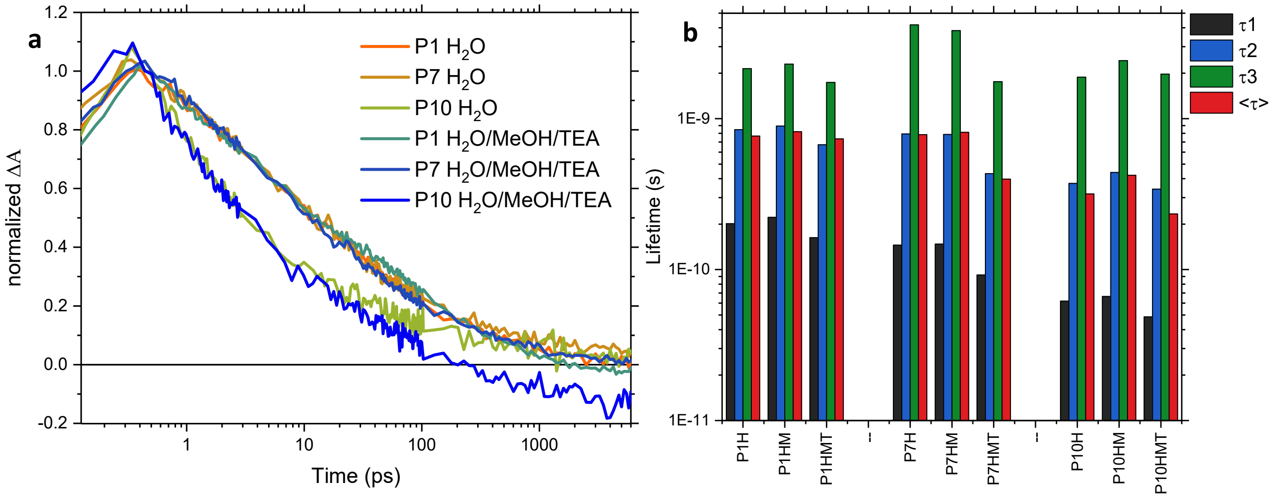

Supplementary Figure 10. a Transient kinetics normalized at 0.5 ps as obtained from suspension of P1, P7, and P10 in aqueous suspension and suspended in the reaction mixture H_2O/MeOH/TEA. Kinetics were probed at 1000 nm upon 355 nm excitation. **b** Fluorescence lifetimes obtained from fitting time-correlated single photon counting decays to a sum of three exponentials, which yield τ_1, τ_2, and τ_3 according to $\sum_{i=1}^{n}(A + B_i \exp(-t/\tau_i))$. <τ> is the weighted average lifetime calculated as $\sum_{i=1}^{n} B_i \tau_i$. Lifetimes are given for all polymers in H_2O (denoted H), H_2O/MeOH (denoted HM), and H_2O/MeOH/TEA (denoted HMT).

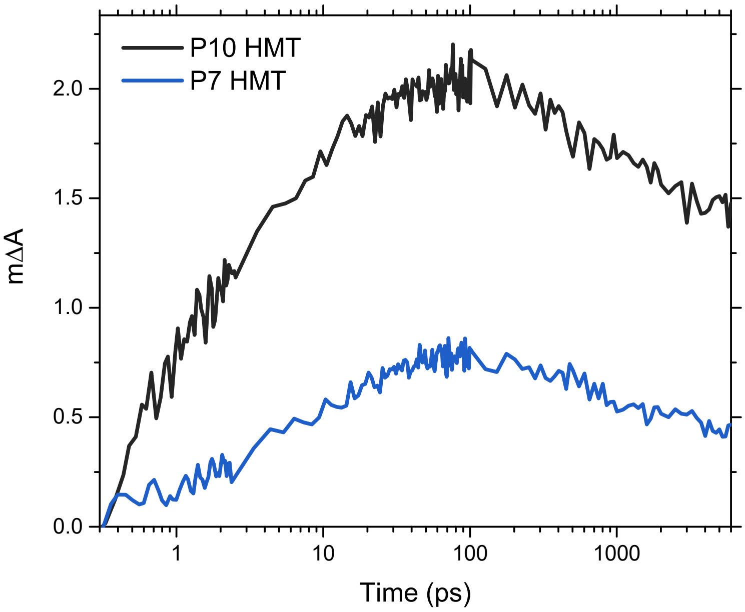

Supplementary Figure 11. Temporal evolution of the 630 nm feature when isolated from the overlapping excitons signals for P7 and P10 suspended in the reaction mixture H_2O/MeOH/TEA. All suspensions were prepared with a polymer concentration of 0.24 g L^{-1} in H_2O and carefully purged with argon before the experiment.

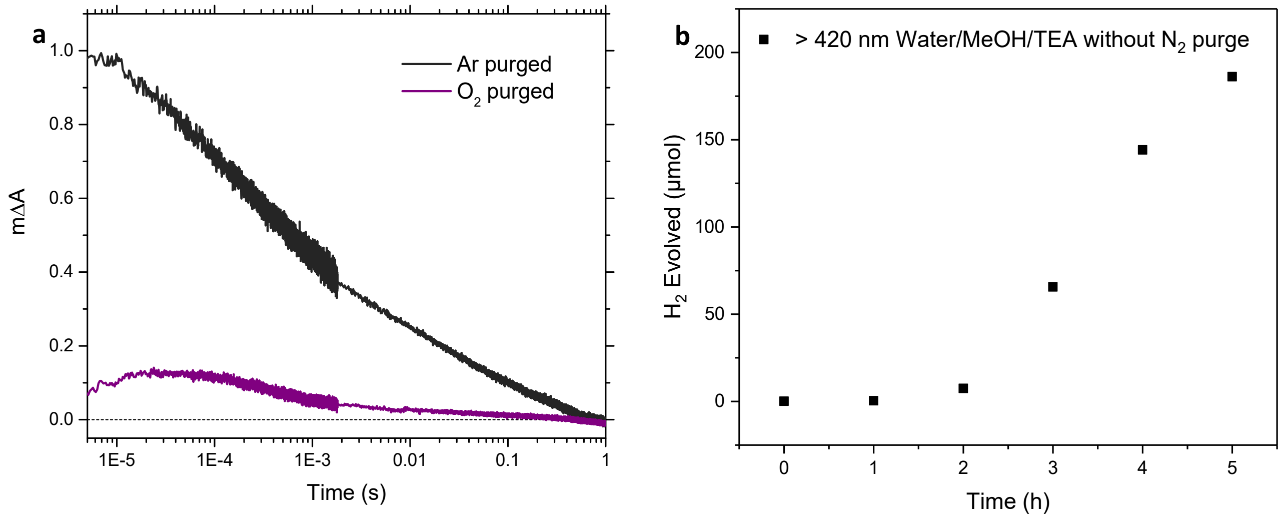

Supplementary Figure 12. Effect of the presence of oxygen in a solvent mixture consisting of equal volumes of H_2O, MeOH, and TEA. **a** Transient kinetics probed at 630 nm for P10 suspended in the reaction mixture. The same suspension was first purged with argon (black trace) and then with oxygen (purple trace). All traces were acquired at an excitation wavelength of 355 nm and an excitation intensity of 1.0 mJ cm^{-2}. **b** Hydrogen evolution of P10 (25 mg) from a H_2O/MeOH/TEA mixture under λ > 420 nm irradiation without N_2 purging the mixture prior to photocatalysis.

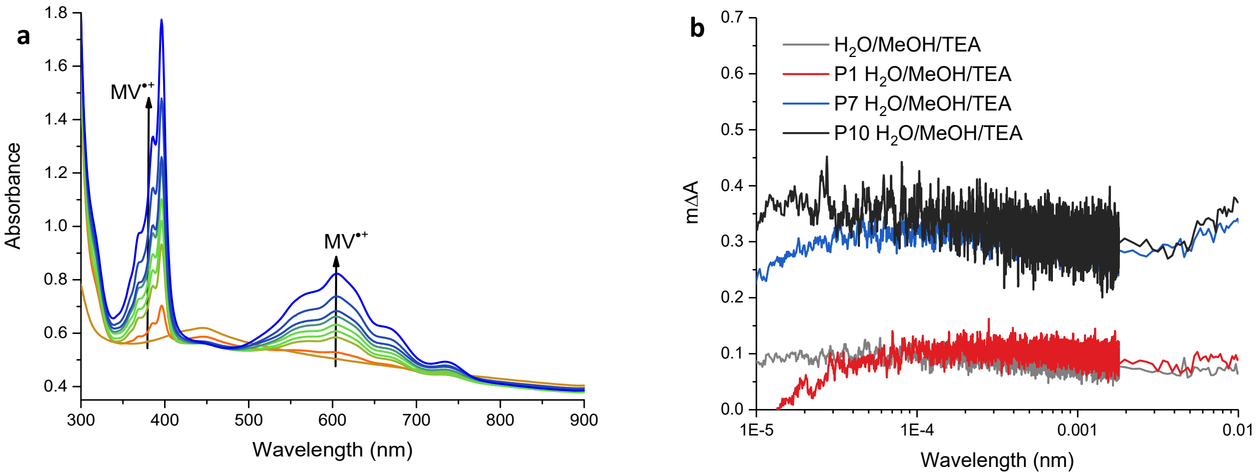

Supplementary Figure 13. a Absorbance spectra after repeated 355 nm photoexcitation of a P10 suspension in H_2O/MeOH/TEA in the presence of 15 mmol L^{-1} methyl viologen. **b** Transient kinetics probed at 600 nm using suspensions with the same composition, but varying the added photocatalyst P1, P7, and P10. Control data in the absence of any photocatalyst is shown for comparison.

We investigate the effect of the electron scavenger methylviologen (*N*,*N*′-dimethyl-4,4′-bipyridinium dichloride) upon addition to our reaction mixture suspensions. **Supplementary Figure 13a** shows the reduction of methylviologen (MV^{2+}) to $MV^{\bullet+}$ upon repeated excitation of a P10 suspension in H_2O/MeOH/TEA, where the newly developed absorption features at below 430 nm and around 600 nm being assigned to the generated $MV^{\bullet+}$ radical cation.[12] The generation of the reduced $MV^{\bullet+}$ radical implies that electron transfer from the polymer to MV^{2+} takes place, suggesting the presence of a reduced polymer species (electron polaron). In **Supplementary Figure 13b**, the kinetics of the $MV^{\bullet+}$ generation as probed at 600 nm are shown. In the absence of any photocatalyst, a certain amount of $MV^{\bullet+}$ is formed through direct electron transfer from TEA. The presence of P1 does not lead to a significantly higher $MV^{\bullet+}$ formation. In contrast, the presence of P7 or P10 results in significantly enhanced $MV^{\bullet+}$ formation (higher ΔA signal amplitude at 600 nm), and the higher ΔA from 10 µs onwards for suggests that electron transfer to MV^{2+} takes place before this timescale. The enhanced methylviologen reduction observed for the more active P7 and P10 together with their more pronounced 630 nm transient peak supports the assignment of this transient species to a polymer centred electron polaron.

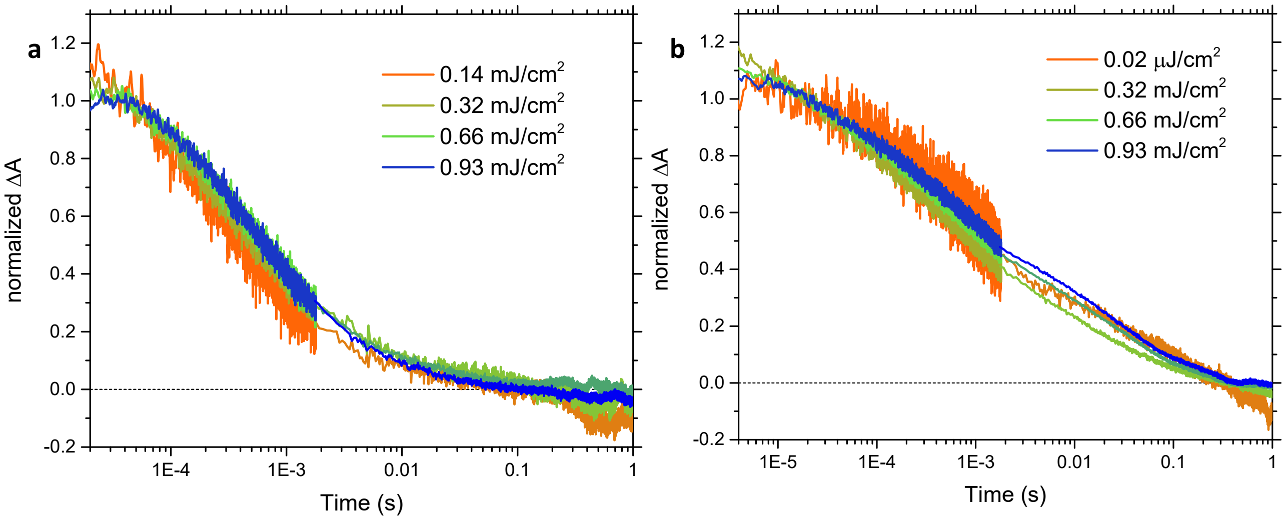

Supplementary Figure 14. Normalized transient kinetics for different excitation fluences, probed at 630 nm for **a** P7 and **b P10** in a solvent mixture consisting of equal volumes of H_2O, MeOH, and TEA upon 355 nm excitation.

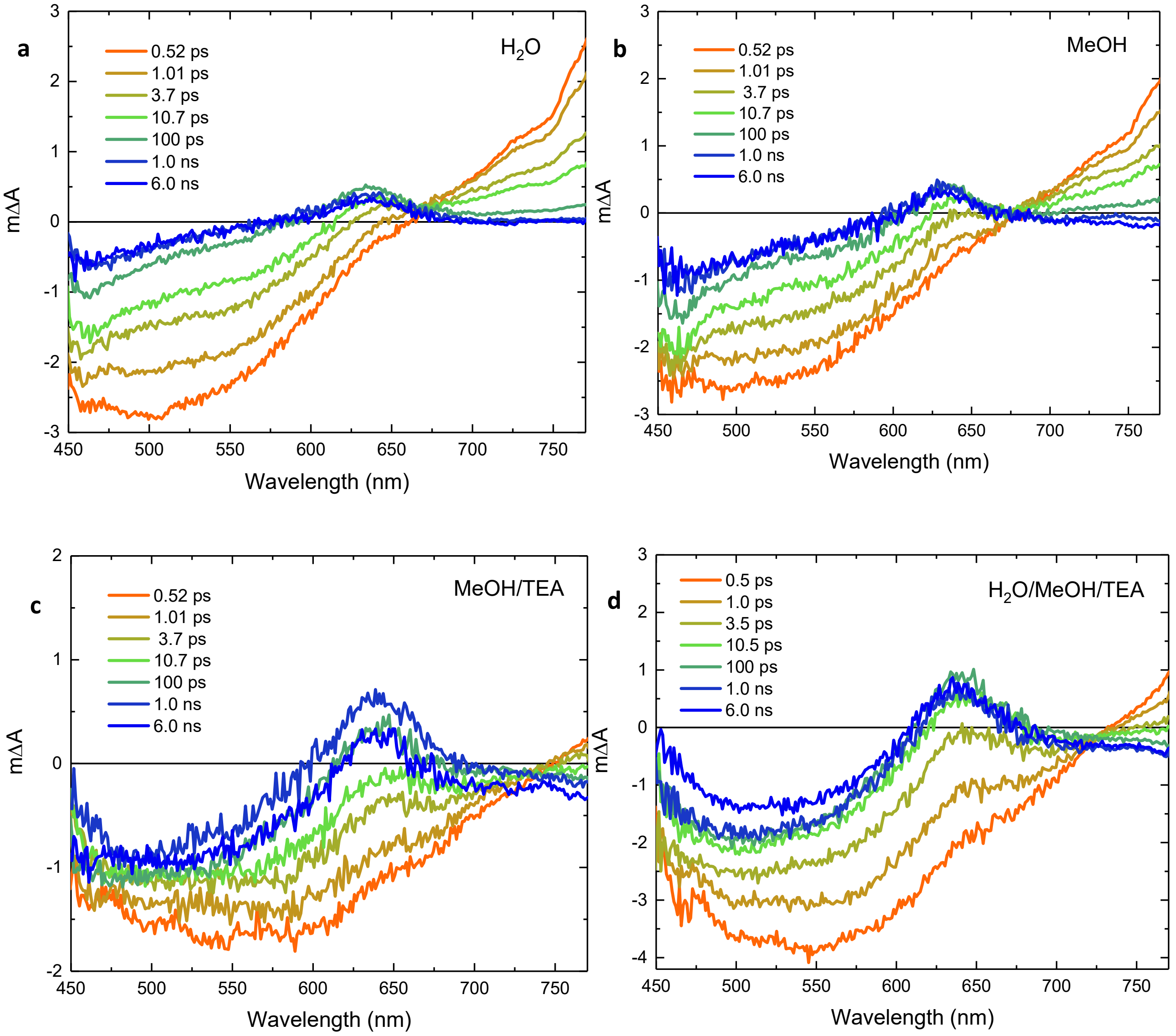

Supplementary Figure 15. Transient absorption spectra obtained from P10 suspended in (**a**) H_2O only, (**b**) MeOH only, (**c**) MeOH/TEA mixture (1:1), and (**d**) H_2O/MeOH/TEA mixture (1:1:1). (**a**) and (**d**) are reproduced here from Figure 2 in the main text for direct comparison. An excitation wavelength of 355 nm and a fluence of 0.08 mJ cm^{-2} were used.

Supplementary Figure 15 shows that P10 suspensions in MeOH only (**Supplementary Figure 15b**) give rise to almost identical transient spectra as P10 suspensions in H_2O only (**Supplementary Figure 15a**). A significant enhancement of the relative amplitude of the 630 nm feature is only observed upon addition of TEA (**Supplementary Figure 15c** and **Supplementary Figure 15d**), which is in line with the much higher photocatalytic activity in the presence to TEA. In keeping with our hydrogen evolution data, these transient data support our interpretation that MeOH does not act as an efficient hole scavenger but rather takes the role of a co-solvent. We note that the lower signal amplitude in the MeOH/TEA experiment (**Supplementary Figure 15c**) can be ascribed to a visibly less well dispersed suspension.

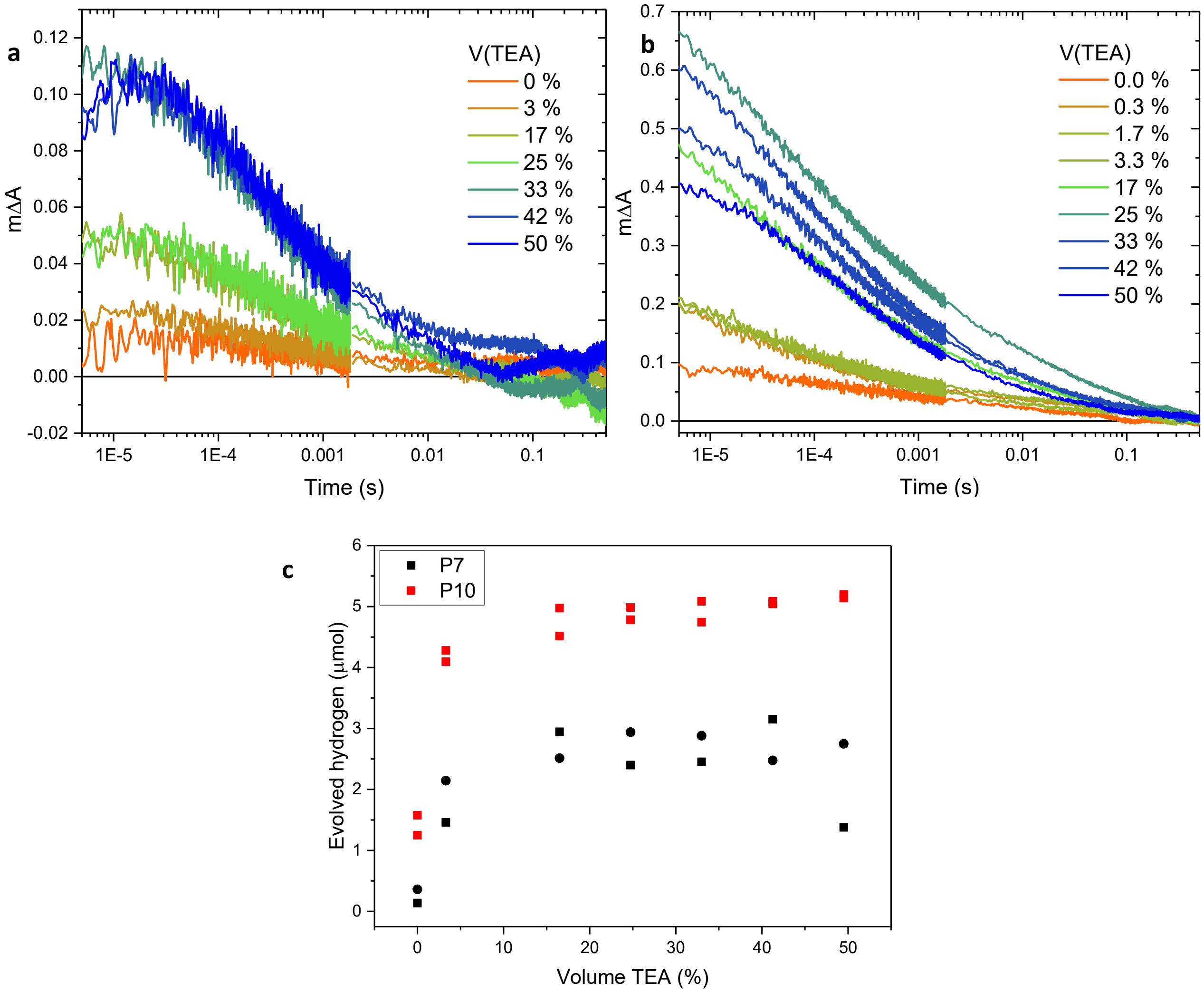

Supplementary Figure 16. Effect of varying the TEA volume fraction while keeping the volume of MeOH constant at 33%, i.e. different TEA volumes were balanced by varying the volume of H_2O. Transient kinetics for **a** P7 and **b** P10 probed at 630 nm upon 355 nm excitation with a fluence of 0.32 mJ cm^{-2}. **c** High-throughput hydrogen evolution experiments, in which the photocatalysts (5 mg) were suspended in the degassed mixture (total volume 5.1 mL), ultrasonicated for 10 min and illuminated for 10 minutes under a visible light panel. The amount of gas was determined using a GC-PDD system calibrated using standards.

The transient kinetics shown in **Supplementary Figure 16a** and **Supplementary Figure 16b** illustrate the influence of different TEA concentrations on the transient kinetics of P7 and P10, as probed at 630 nm (note that the TEA volume fraction in all other transient measurements was 33%). We observe an increasing transient signal up to 33% TEA for P7 and up to 25% TEA for P10, consistent with higher polaron yields in the presence of more TEA and in good agreement with the H_2 evolution data in **Supplementary Figure 16c**. While the signal amplitude for P7 saturates at 33% TEA, a decrease in amplitude is observed for P10 above 25% TEA, which we ascribe to an increasing agglomeration of P10 particles beyond this TEA

concentration. Since the MeOH volume was kept constant at 33% for this series of experiments, these results suggest that the polaron yield (*i.e.*, the amplitude of the 630 nm signal on this timescale) is modulated by the concentration of TEA, rather than by the concentration of MeOH, supporting the idea that MeOH does not act as an efficient hole scavenger for either P7 or P10, even in the presence of TEA.

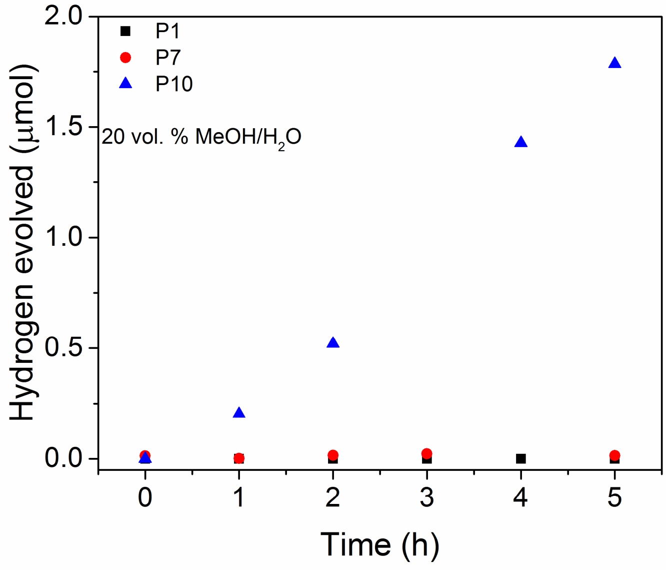

Supplementary Figure 17. Hydrogen evolution of P1, P7, and P10 (25 mg) from water/methanol (20 vol. %) mixture under λ > 420 nm irradiation.

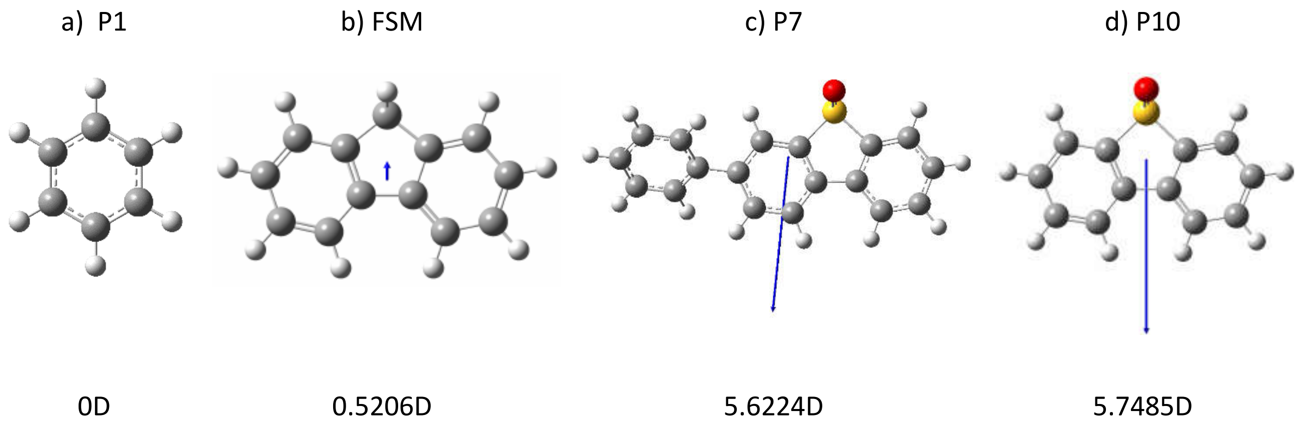

Supplementary Figure 18. Calculated dipole moments for monomers of **a** P1 **b** FSM **c** P7 **d** P10. This was done using DFT, B3LYP/6-311G(d,p) with Gaussian 16.

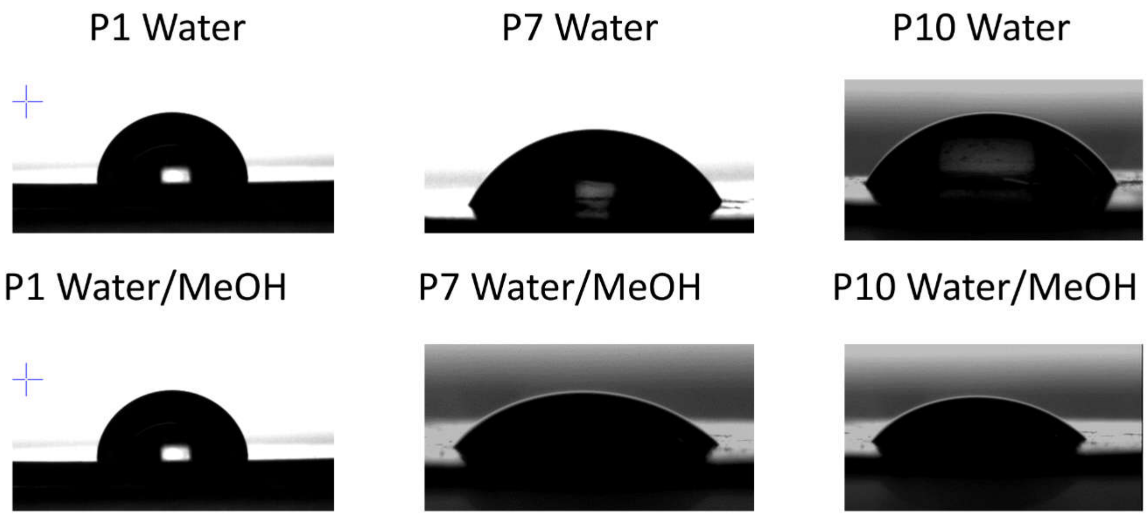

Supplementary Figure 19. Images of contact angle measurement of P1, P7, and P10. The polymers were pressed into pellets with a press at 7 bar pressure between two 1.3 cm dies. Ultrapure water and 1:1 mixtures of water and methanol were used for the measurements. For all materials the contact angles against water/methanol/triethylamine were too low to be measured. We note that the contact angles with water cannot be directly compared to those with water/methanol due to differences in surface tension between the two solvents.

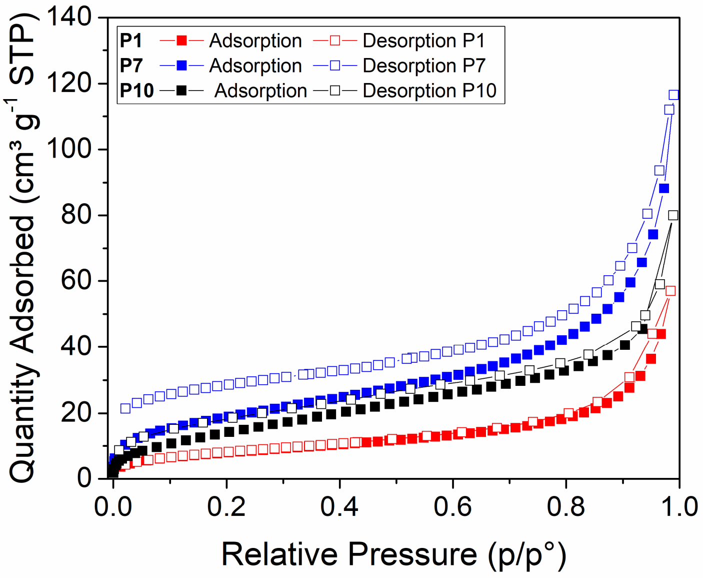

Supplementary Figure 20. Nitrogen sorption isotherms for polymer P1, P7 and, P10 measured at 77.3 K and up to 1 bar (desorption curves shown as open symbols). The BET surface areas were calculated to be SA_{BET} = 29 $m^2 g^{-1}$ for P1, SA_{BET} = 69 $m^2 g^{-1}$ for P7, and SA_{BET} = 56 $m^2 g^{-1}$ for P10.

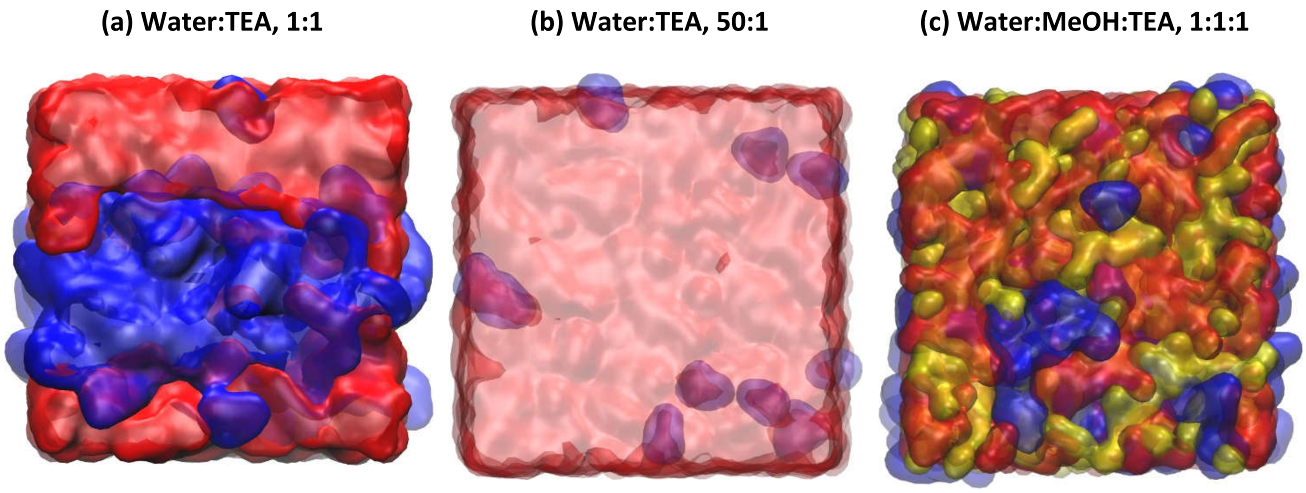

Supplementary Figure 21. Snapshots of the end of a 20 ns simulation of mixtures of different solvents which have been randomly intermixed before being simulated. **a** We see a high degree of phase separation between water and TEA, **b** Dramatically reducing the concentration of TEA allows mixing when individual TEA molecules can no longer interact with each other. **c** Methanol mixes well with water but does not prevent segregation of the TEA phase. Red represents water, blue triethylamine and yellow represents methanol. All ratios are the ratio of molar volumes.

a) FSM b) P7 c) P10

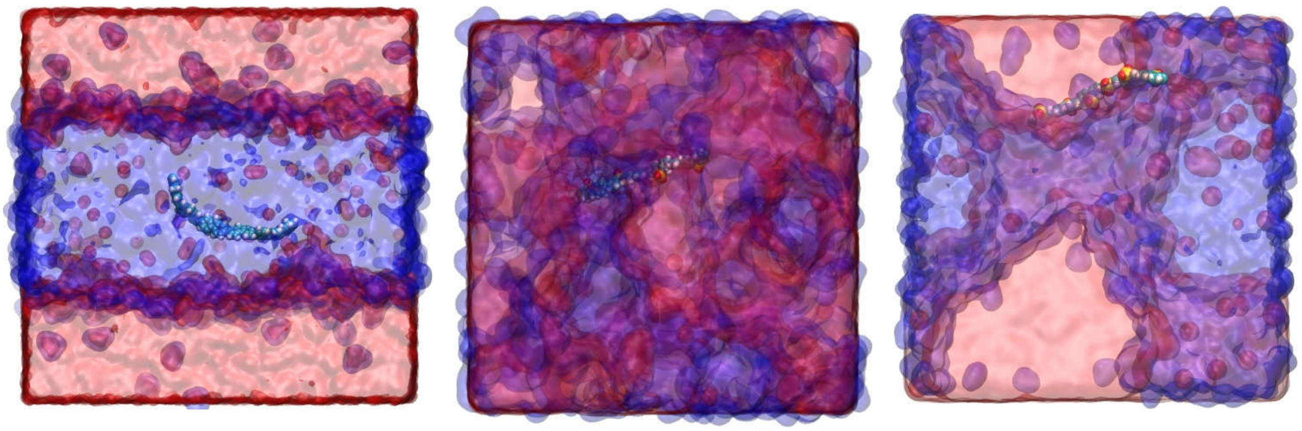

Supplementary Figure 22. Snapshots of oligomers after 10 ns of simulation of **a** FSM **b** P7 and **c** P10 in a 1:1 mix of water and TEA (red = water; blue = TEA). The sulfone tips aligning into the water phase mean that the interface curls to match the natural curve of the oligomers curvature when the dipoles align. Red represents water, blue triethylamine and oligomers are shown by van-der-Waals spheres coloured by atom, with sulfur and oxygen being yellow and red respectively. It can be seen that FSM resides in the triethylamine and that P7 and P10 at the interface. P7 appears to promote less phase segregation.

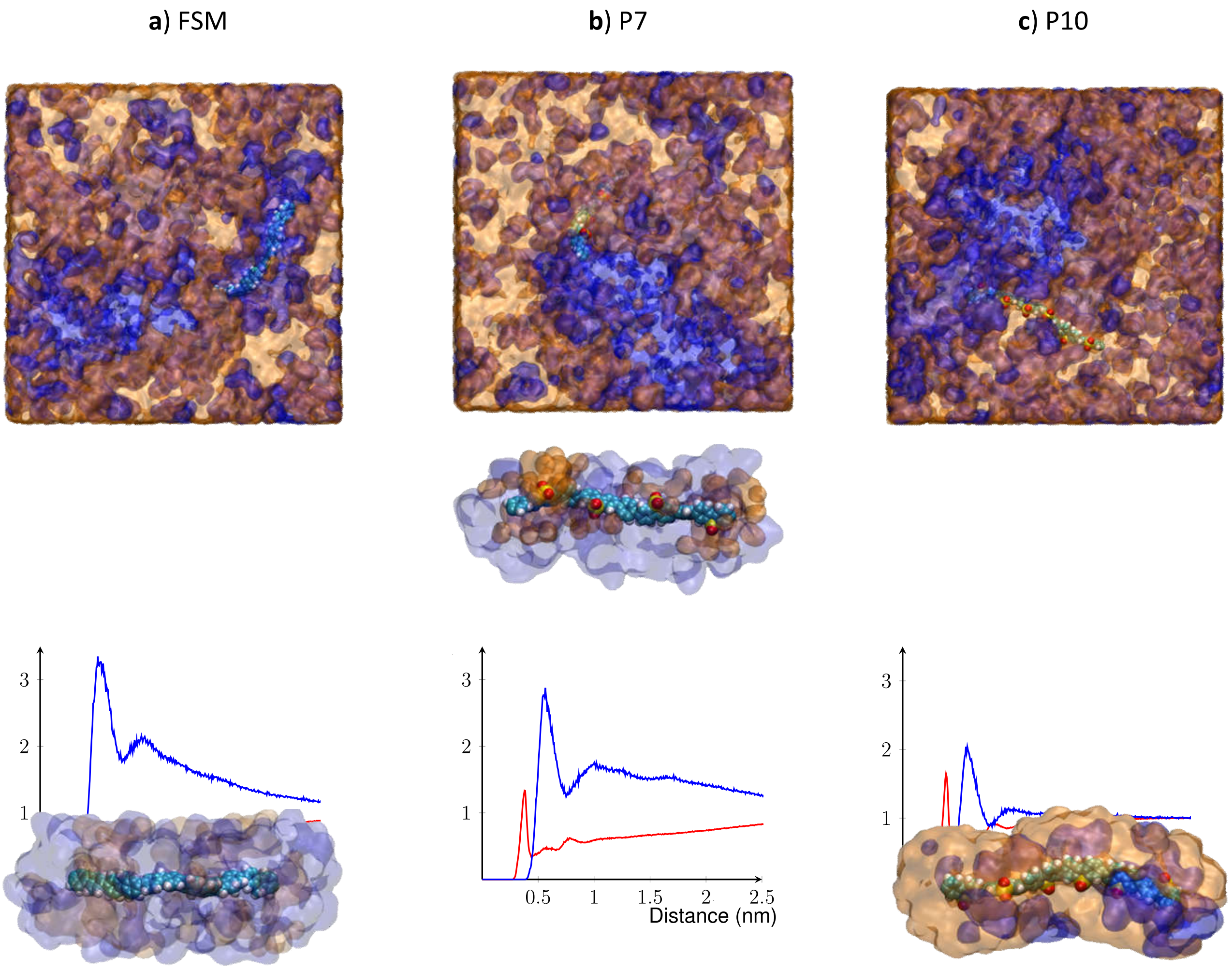

Supplementary Figure 23. Effect of including methanol in the system: non-sulfone polymers continue to exist in TEA-rich environments and sulfone polymers contain themselves in a more water-rich environment. Top shows a snapshot with of the whole box with the TEA phase shown in blue and the water/methanol phase shown in orange; Middle shows the local (within 1 nm) environment of the polymer for clarity; bottom shows the Radial Distribution functions for the polymer with TEA (blue) and water (red) for **a** FSM **b** P7 and **c** P10.

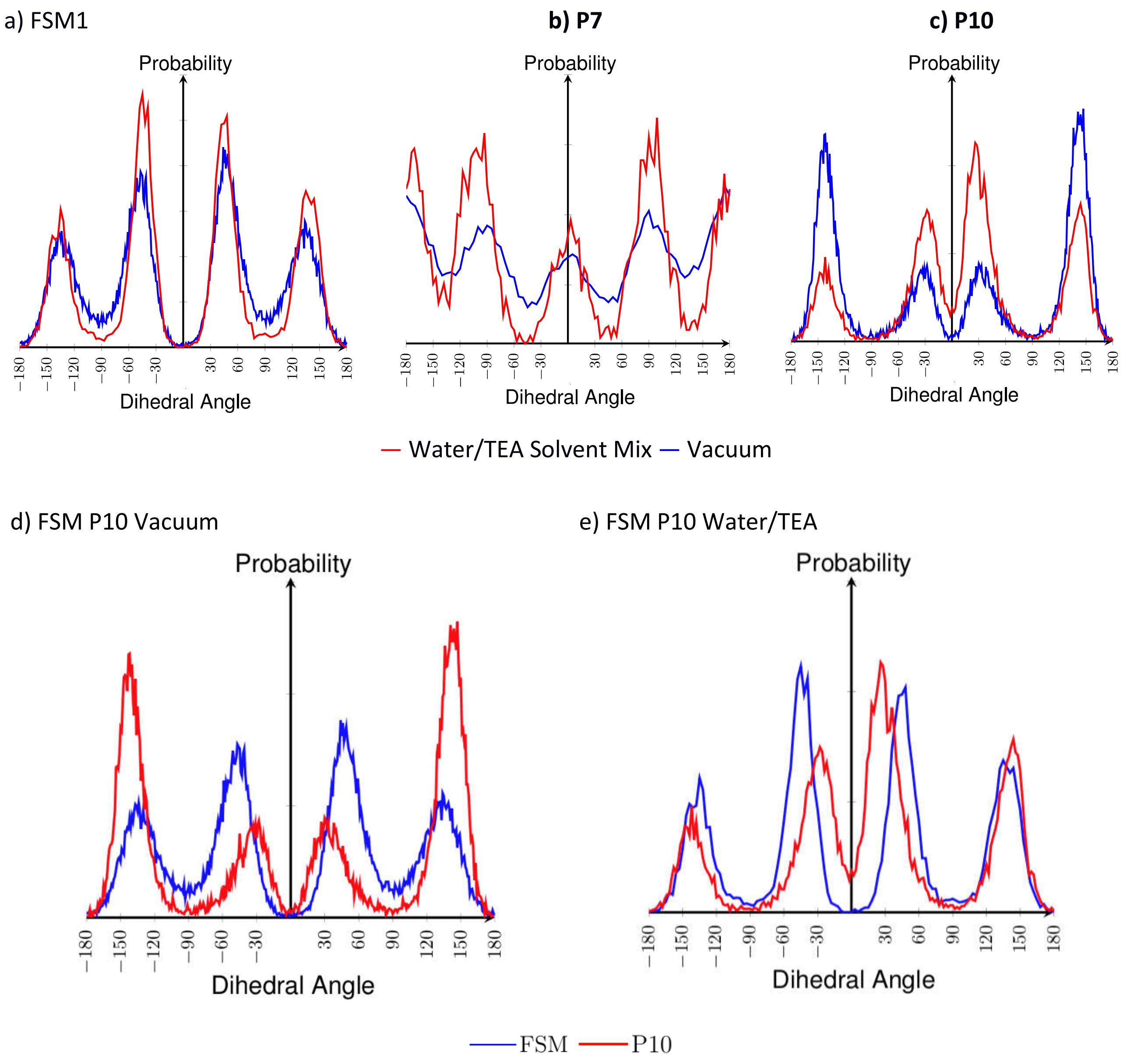

Supplementary Figure 24. Shows the probability distributions of intermonomer dihedral angles found from MD simulations of dimers of **a** FSM **b** P7 and **c** P10 in both vacuum (blue) and water/TEA 1:1 mix (red). It can be seen that presence of the solvent makes no significant difference to FSM which slightly favours aligning with the bridging carbon atoms on the same side (dihedral angles closer to 0 than 180°) in both solution and vacuum. For P10, the introduction of the solvent causes a dramatic realignment from favouring anti-alignment of dipoles in vacuum to strongly favouring alignment of dipoles in the reaction mix. This alignment is necessary if all sulfones groups are to be embedded in the water phase. P7 does not show realignment because water domains are smaller and dragged around with the sulfone group. In addition, direct comparisons of the distribution of dihedral angle for the FSM (blue) and P10 (red) oligomers are shown in **d** for vacuum and **e** for the TEA/water mix.

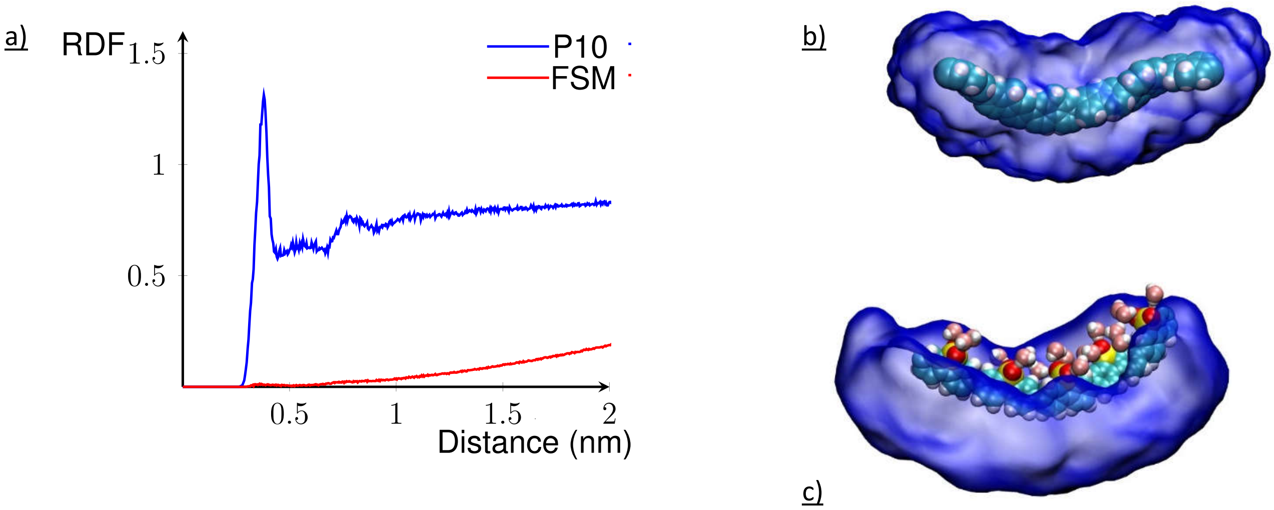

Supplementary Figure 25. This figure demonstrates the key effect of the sulfone group, to ensure a water shell surrounds the oligomer. **a** The radial distribution function (RDF) between the sulfone group and water molecules for P10 (blue) and FSM (red), showing the presence of a structured water shell around the sulfone group and the absence of any alignment of water around the bridging carbon of a fluorene oligomer. The distance is measured between centre of mass of the water and the bridging (sulfur or carbon) atom. **b** and **c** show all TEA molecules within 1 nm as a blue surface and all water molecules within 0.6 nm are shown explicitly in pink around FSM and P10 respectively, displayed explicitly and coloured by atom type. The simulations are for hexamers of FSM and P10 in a water:TEA solvent mix.

a)

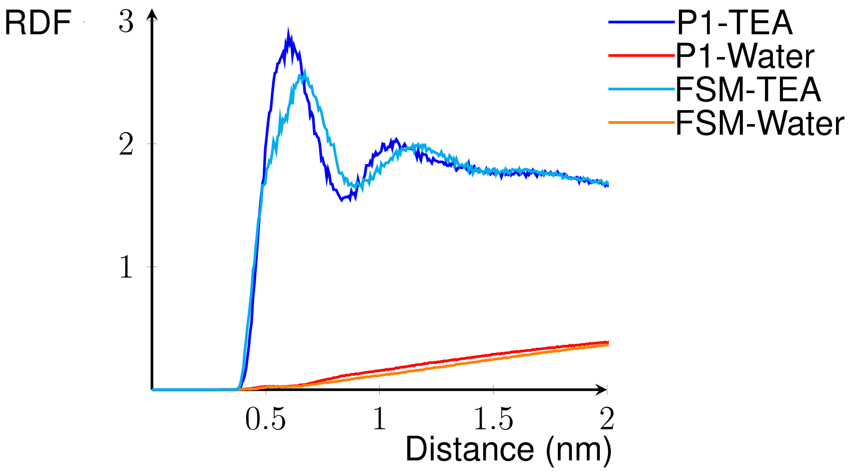

b)

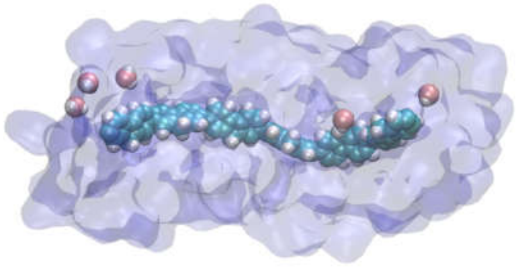

c)

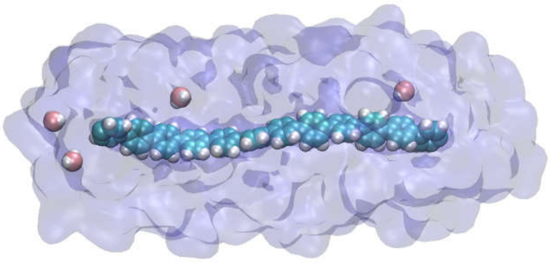

Supplementary Figure 26. This figure demonstrates that with respect to the solvent environment the oligomers of P1 and FSM behave the same. **a** shows the Radial distribution function (RDF) of the oligomers with both TEA and water. Since both polymers reside in majority TEA phase the RDF with water is very small. **b** and **c** show all TEA molecules within 1 nm as a blue surface and all water molecules within 0.6 nm are shown explicitly in pink around P1 and FSM respectively, displayed explicitly and coloured by atom type.

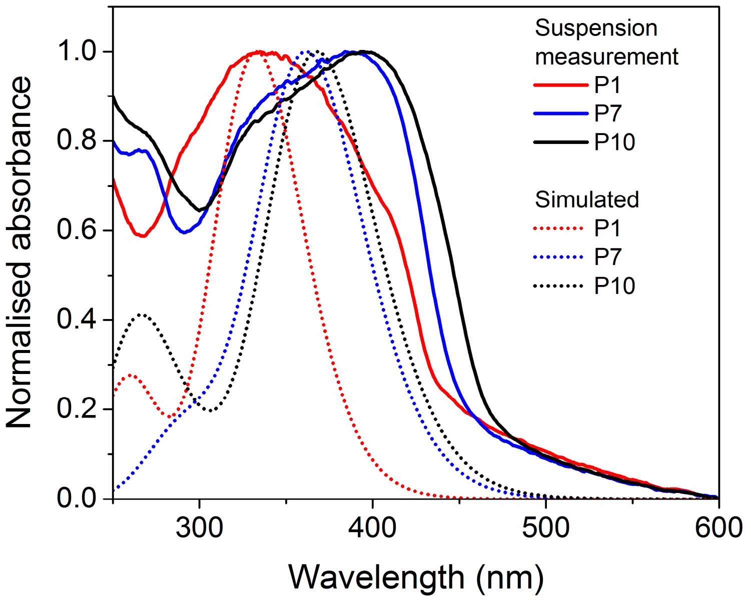

Supplementary Figure 27. Comparison of measured UV-vis spectra of dispersions in water with scattering and reflectance subtracted (solid lines), with absorption coefficients calculated for oligomers of the polymers (hexamer of P1, dimer of P7, trimer of P10) using time-dependent density functional theory (B3LYP/6-31G*) (dashed lines).

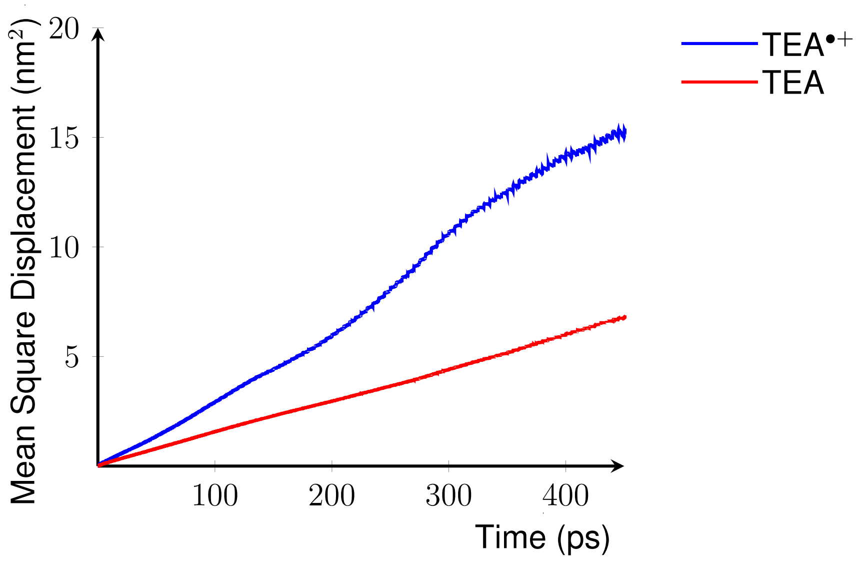

Supplementary Figure 28. Mean square displacement plots for TEA and $TEA^{•+}$, which are used to estimate the diffusion constant of those species in a bi-layer system containing water, TEA and $TEA^{•+}$ (initially in the TEA phase). Yields diffusion constants of 2.46 ± 0.3 nm^2 ns^{-1} and 6.27 ± 0.64 nm^2 ns^{-1} for TEA and $TEA^{•+}$ respectively calculated using the gmx msd tool which utilizes an Einstein relation. This increase in diffusion constant is the result of the TEA^{+} migrating into the water phase.

Supplementary Figure 28 considers the solvent environment to be fixed on the time scale of the transient optical experiments. To test this, we carried out MD simulations of the diffusion of TEA molecules and $TEA^{•+}$ radicals in a system containing water and TEA, obtaining diffusion coefficients of around 1.5 and 1 nm^2 ns^{-1} for TEA and $TEA^{•+}$, respectively. The charged radical diffuses slightly slower: it is driven towards the water phase where it is stabilized. The diffusion coefficient is too low for any significant solvent redistribution to happen on the <1 ns time scale of the ultrafast spectroscopic measurements. We can therefore rule out the dynamics of TEA molecules or radicals as an explanation for the different behaviour of the three polymers on ultrafast time scales. Molecular diffusion will still be relevant on the longer time scales for hydrogen evolution.

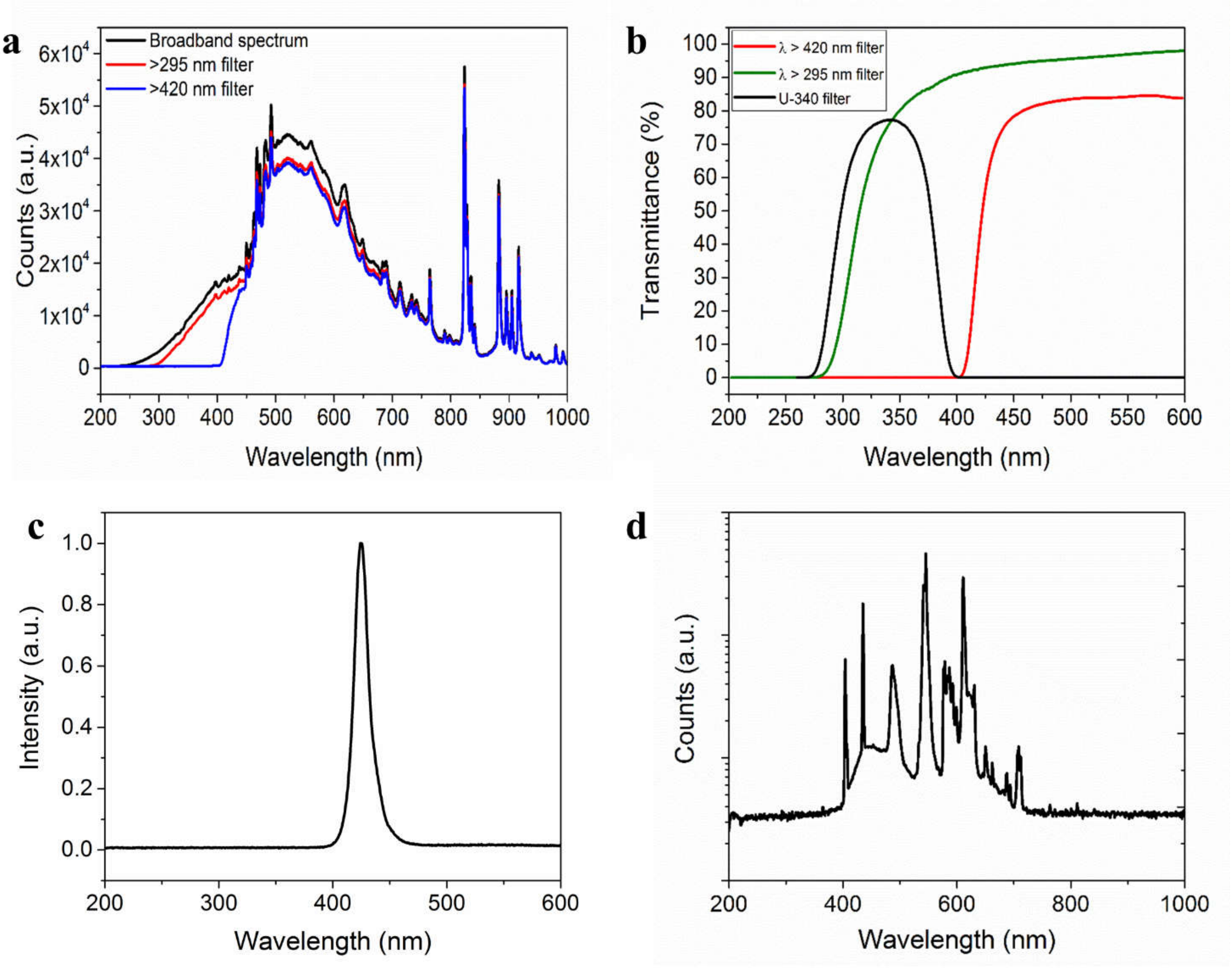

Supplementary Figure 29. **a** Spectral output of the Xe light source **b** Transmittance characteristics of the > 420 nm, > 295 nm, and U-340 filter **c** Output of the 420 nm LED **d** Output of the visible-light panel used for hydrogen evolution experiments performed with varied amounts of triethylamine (TEA) in the water/methanol/TEA mixture.

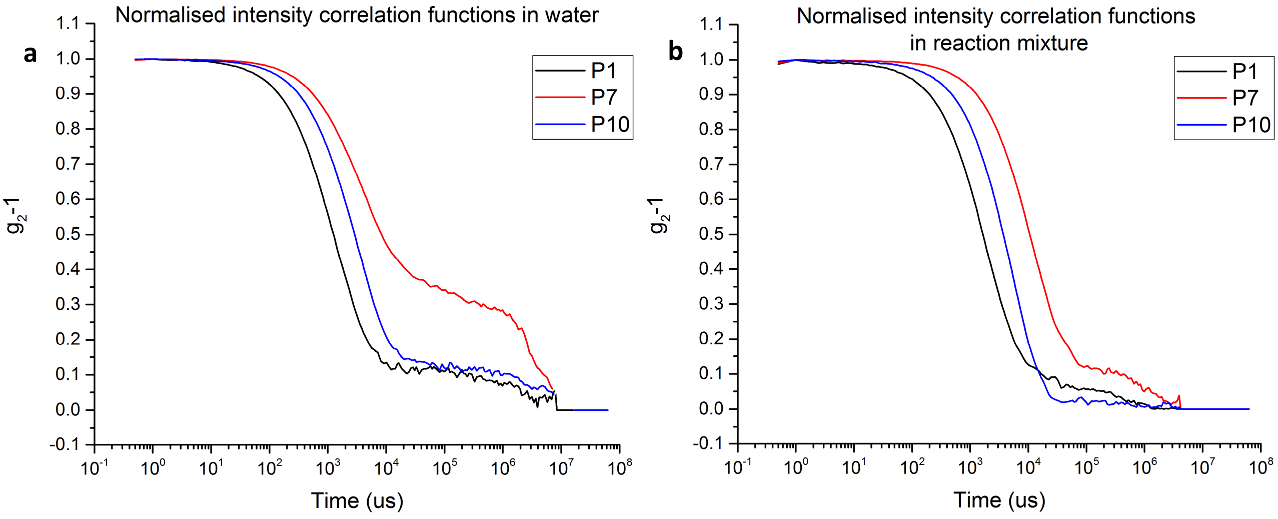

Supplementary Figure 30. Representative normalized intensity correlation functions from dynamic light scattering measurements for P1, P7 and P10 dispersed in **a** water and in **b** reaction mixture.

a) FSM b) P7 c) P10

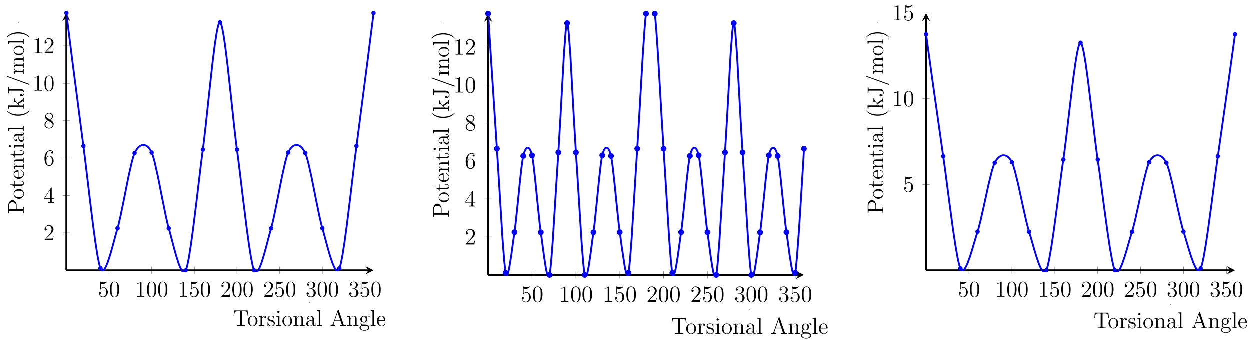

Supplementary Figure 31. Torsional potentials. Results of DFT calculations on dimers in the case of FSM and P10 and a monomer of P7 where the dihedral angle has been scanned. Potential minima occur at 39°, 38°, 38° with a periodicity of 90° for FSM, P7 and P10 respectively. The MD torsion potential is then parameterized to as close as possible match the DFT calculation. Parameters used are shown in Supplementary Tables 8-10.

Supplementary tables

'**Supplementary Table 1.** Peak assignments of ^{13}C NMR chemical shifts for **P10**.

δ_{iso} / ppm	^{13}C assignments
117	-SC***C***H-
121	-SCCH***C***Br-
125	-SCC***C***H-
130	-SCCH***C***-, -SCCCH***C***H-
137	-SC***C***-
139	-S***C***-

Supplementary Table 2. Mean particle sizes for P1, P7 and P10, dispersed in water and reaction mixture (H_2O/MeOH/TEA).

Mean particle size / Polymer	Water / nm	Reaction mixture / nm
**P1**	**534** ± 47	**431** ± 39
**P7**	**913** ± 76	**2199** ± 103
**P10**	**647** ± 11	**1507** ± 314

Supplementary Table 3. B3LYP/DZP/COSMO (ε_r 80.1) predicted potentials of the different polymers in the presence of water. All values in volt. These potentials correspond to those plotted in Fig. 5c and 5d in the main text.

	**P1**[a]	**P7**[a]	**P10**
**EA**	-2.33	-1.81	-1.59
**IP***	-2.23	-1.63	-1.44
**EA***	0.79	1.06	1.28
**IP**	0.88	1.24	1.43

[a] values taken from our previous work.[1]

Supplementary Table 4. B3LYP/DZP/COSMO (ε_r 2.38) predicted potentials of the different polymers in the presence of TEA. All values in volt. These potentials correspond to those plotted in Fig. 5c and 5d in the main text.

	**P1**	**P7**	**P10**
**EA**	-2.88	-2.23	-1.93
**IP***	-1.65	-1.08	-0.82
**EA***	0.12	0.63	0.88
**IP**	1.35	1.78	2.00

Supplementary Table 5. B3LYP/DZP/COSMO predicted potentials of the different solution half reactions in water and TEA using $TEAH^+$ written following convention as reductions. All values in volt.

Solution half-reaction	E	
	water	TEA
$TEAH^+$ (sol) + e^- -> 1/2 H_2 (g) + TEA (l/sol)	-0.78	0.26
TEA^{*+} (sol) + e^- -> TEA (l/sol)	0.66	1.39
TEAR (sol) + $TEAH^+$ (sol) + e^- -> 2 TEA (l/sol)	0.59	1.50
DEA (sol) + AcO (sol) + $TEAH^+$ (sol) + e^- -> TEAR (sol) + TEA (l/sol) + H_2O (l/sol)	-2.22	-1.01
DEA (sol) + AcO (sol) + 2 $TEAH^+$ (sol) + 2 e^- -> 3 TEA (l/sol) + H_2O (l/sol)	-0.82	0.25

TEA = triethylamine, TEAR = triethylamine radical ($Et_2(CH_3CH)N^*$), DEA = diethylamine, AcO = acetaldehyde

Supplementary Table 6. B3LYP/DZP/COSMO predicted solvation energies of the different solution species in water and TEA where the solvation energy is defined as the difference in energy between a molecule in vacuum and embedded in a dielectric continuum with dielectric permittivity ε_r. All values in electronvolt.

	**$U_{solvation}$**	
	**Water** (ε_r 80.1) / eV	**TEA** (ε_r 2.38) / eV
Water	-0.29	-0.13
TEA^{*+}	-2.22	-1.08
TEA	-0.08	-0.03
$TEAH^+$	-2.30	-1.12
TEAR	-0.08	-0.03
AcO	-0.20	-0.09
DEA	-0.12	-0.05

TEA = triethylamine, TEAR = triethylamine radical ($Et_2(CH_3CH)N^*$), DEA = diethylamine, AcO = acetaldehyde

Supplementary Table 7. B3LYP/DZP/COSMO predicted reaction energies (U_r) and free energies (G_r) for relevant (half) reactions in vacuum, TEA (ε_r 2.38) and water (ε_r 80.1). All values in electonvolt.

	U_r / eV			G_r / eV	
	**vacuum**	**TEA**	**water**	**TEA**	**water**
**$TEAH^+$ (sol) + e^- -> 1/2 H_2 (g) + TEA (l/sol)**	-5.34	-4.26	-3.12	-4.70	-3.66
**TEA^{*+} (sol) + e^- -> TEA (l/sol)**	-7.11	-6.07	-4.97	-5.83	-5.10
**TEAR (sol) + $TEAH^+$ (sol) + e^- -> 2 TEA (l/sol)**	-7.01	-5.93	-4.78	-5.94	-5.03
**DEA (sol) + AcO (sol) + $TEAH^+$ (sol) + e^- -> TEAR (sol) + TEA (l/sol) + H_2O (l/sol)**	-4.41	-3.36	-2.24	-3.42	-2.22
**DEA (sol) + AcO (sol) + 2 $TEAH^+$ (sol) + 2 e^- -> 3 TEA (l/sol) + H_2O (l/sol)**	-11.42	-9.29	-7.01	-9.40	-7.25
**TEA^{*+} (sol) + TEA (l/sol) -> TEAR (sol) + $TEAH^+$ (sol)**	-0.10	-0.14	-0.19	0.11	-0.07

TEA = triethylamine, TEAR = triethylamine radical ($Et_2(CH_3CH)N^*$), DEA = diethylamine, AcO = acetaldehyde

The large differences in the solvation energies of charged species (e.g. TEA^+) when dissolved in TEA or water, visible in **Supplementary Table 6**, translate in similarly large differences in the reaction (free) energies for the different half-reactions, as can be seen here in **Supplementary Table** 7. For total reactions, where there are equal amounts of charged species on either side of the reaction arrow, e.g. the deprotonation of TEA^+, the effect of solvation is much smaller. Calculations using the PCM+SMD[2,3] solvation model yield very similar solvation energies to those in Table S12 other than a subtle inversion of the solvation energies of TEA/TEAR in water and TEA, probably related to an improved description of non-electrostatic contributions to the solvation energies for these neutral species with small dipoles, which has a very minor effect on the reaction energies and potentials since those are dominated by the solvation energies of the charged species.

Supplementary Table 8. Shows the parameters found by fitting Ryckaert-Bellemans functions $V_{rb}(\phi_{ijkl}) = \sum_{n=0}^{5} C_n (\cos(\psi))^n$ with $\psi = \phi - 180°$ to the dihedral profile found using DFT (Supplementary Figure 29). φ is the dihedral angle between C3, C4 on one monomer and C4, C3 on the next monomer with φ defined as 0 when dipole moments align. All constants are in units of kJ mol^{-1}.

	C0	C1	C2	C3	C4	C5
**FSM1**	6.6779	1.21407	-32.3384	2.79435	14.5608	-5.27441
**P7**	7.77182	-0.80391	-31.3923	9.27213	10.0503	-9.23086
**P10**	9.49828	1.24041	-25.2547	-6.97006	0.366191	8.4949

Supplementary Table 9. Partial charges on neutral[4] and cationic triethylamine used in the molecular dynamics simulations.

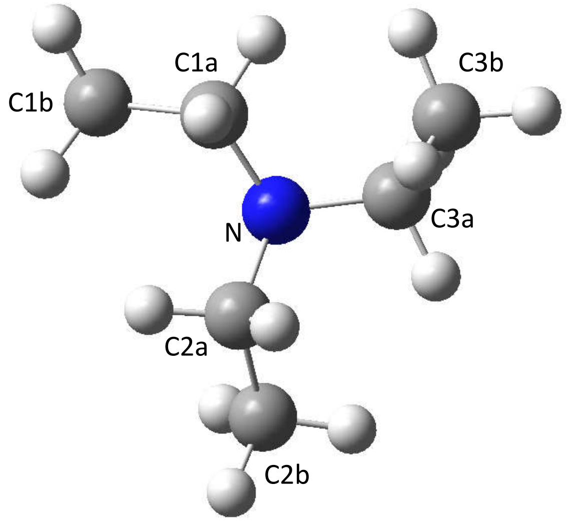

Atom	TEA (units of electronic charge)	TEA+ (units of electronic charge)
N	-0.63	-0.0105
C1a	0.09	0.1101
H1a1	0.06	0.0819
H1a2	0.06	0.0729
C1b	-0.18	-0.1497
H1b1	0.06	0.0750
H1b2	0.06	0.0670
H1b3	0.06	0.0794
C2a	0.09	0.1179
H2a1	0.06	0.0800
H2a2	0.06	0.0709
C2b	-0.18	-0.1578
H2b1	0.06	0.0690
H2b2	0.06	0.0762
H2b3	0.06	0.0082
C3a	0.09	0.1046
H3a1	0.06	0.0743
H3a2	0.06	0.0840
C3b	-0.18	-0.1487
H3b1	0.06	0.0762
H3b2	0.06	0.0796
H3b3	0.06	0.0669

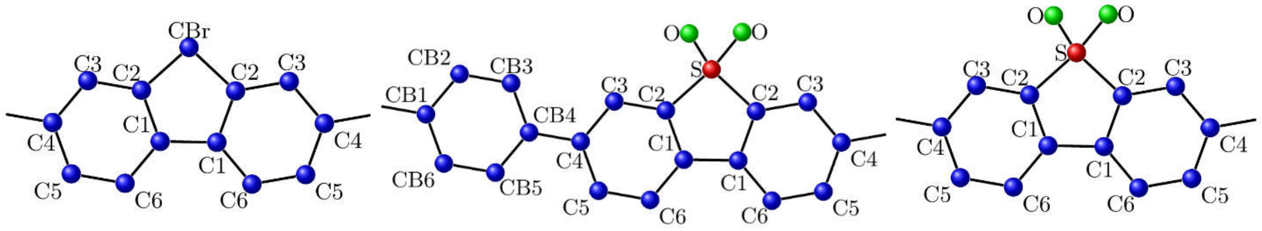

Supplementary Table 10. Partial charges on monomers of the three polymers used in the molecular dynamics simulations. They were found by calculating partial charges using the CHELPG population for oligomers in different conformations. The partials charges found were then averaged across conformation and monomer (to average edge effects). Minor alterations were then made to ensure a) symmetry was upheld and the b) each monomer was charge neutral, in line with other methods used in the literature[5] (as opposed to the alternative of distinguishing between edge monomers and central monomers[6]).

Atom	**FSM1** Partial Charge (in units of electric charge)	**P7** Partial Charge (in units of electric charge)	**P10** Partial Charge (in units of electric charge)
C1	0.034	0.062	0.068
C2	-0.041	-0.181	-0.177
C3	-0.154	-0.041	-0.036
H3	0.093	0.118	0.117
C4	0.048	0.036	0.021
C5	-0.118	-0.063	-0.142
H5	0.087	0.09	0.117
C6	-0.144	-0.139	-0.142
H6	0.109	0.118	0.085
S	**N/A**	1.174	1.166
O	**N/A**	-0.6	-0.583

CB1	**N/A**	0.025	**N/A**
CB2	**N/A**	-0.097	**N/A**
HB2	**N/A**	0.091	**N/A**
CB3	**N/A**	-0.097	**N/A**
HB3	**N/A**	0.091	**N/A**
CB4	**N/A**	0.025	**N/A**
CB5	**N/A**	-0.097	**N/A**
HB5	**N/A**	0.091	**N/A**
CB6	**N/A**	-0.097	**N/A**
HB6	**N/A**	0.091	**N/A**
Cbr	0.192	**N/A**	**N/A**
HBr	-0.01	**N/A**	**N/A**

Supplementary methods

1. Dynamic light scattering methodology

The Brownian motion of each sample was characterized using an intensity correlation function g_2, which compares the intensity at the detector at initial time t to the intensity at later times τ:

$$g_2(\tau) = \frac{\langle I(t).I(t+\tau)\rangle}{\langle I(t)\rangle^2} = 1 + |g_1(\tau)|^2$$

Here g_1 is the electric field correlation function, $I(t)$ the intensity at time t and the angular brackets represent the temporal average[7]. The field function can be expressed as the linear combination of a series of exponential terms:

$$g_1 = \sum_i A_i e^{-\Gamma_i \tau}$$

where A_i and Γ_i are respectively the amplitudes and decay rates for each exponential term in the series. Each term in the summation correlates to scattering from particles of a different size.[8] All data were fit with single, double and triple exponentials (i=1-3) using OriginPro 2017. In all cases, single exponentials produced poor fits whilst triple exponentials overfitted the data. The data were also fit to sums of stretched exponentials, but this was found to have no significant effect on the extracted particle size. As the inclusion of stretch factors increases the number of fit parameters, a two-term normal exponential model was used to prevent overfitting of the data.[8]

The mean hydrodynamic diameter of the dispersed particles (d) was then calculated from each correlation function decay rate using:[8]

$$d = \frac{1}{\Gamma}\frac{k_B T}{3\pi\eta}\frac{16\pi^2 n^2}{\lambda^2}\sin^2\left(\frac{\theta}{2}\right)$$

Here k_B is Boltzmann's constant, T is temperature, η is the dynamic viscosity of the liquid medium, n is the solvent refractive index, λ is the incident laser wavelength and θ is the measured scattering angle. This equation was derived from the Stokes-Einstein relation for spherical particles along with the definition of the scattering vector magnitude q in our setup:

$$q = \frac{4\pi n}{\lambda} \sin\left(\frac{\theta}{2}\right)$$

and the relation between decay rate and translational diffusion coefficient $\Gamma = q^2 D$. Extracted particle diameters from repeat measurements were then averaged to produce the values in **Supplementary Table 2**. The standard deviation of the spread of particle sizes was used to calculate the associated errors.

2. Calculations of static dipole moments

For all molecular dynamics simulations we employed a fluorene polymer, referred to as FSM1, in place of P1. This was in order to more directly isolate the effect of the presence of the sulfone group.

Static dipole moments were calculated from ground state DFT calculations. All ground state DFT calculations were performed using Gaussian 16 employing the B3LYP functional and the 6-311G(d,p) basis set.

3. Atomistic molecular dynamics

Atomistic molecular dynamics (MD) simulations were carried out in the GROMACS package.[9–12] Forcefields, resulting torsional potentials and partial charge distributions are given below. The simulation workflow is illustrated below.

In fully atomistic MD simulations, state-of-the-art simulations deal with hundred of thousands of atoms with production run reaching 100 ns. Interchain interactions with itself (in other word with its periodic images) must be avoided. Therefore, a buffer of solvent of the order of the cut-off used in the force fields for non-bonded interactions must be used. One can easily see how the size of the simulation is scaling with the length of the oligomer. Larger boxes can be studied at the expenses of the length of the simulation; however as a result, the statistics will be poor and the ergodic theorem won't hold. Polymers are therefore especially challenging because of their size and the breadth of their relaxation times. As a consequence, it is common practice to simulate oligomers instead of polymers in fully atomistic MD simulations. Here, we use oligomers containing twelve aromatic rings (hexamers of FSM and P10, tetramers of P7) in mixtures of water and TEA, or of water, methanol and TEA.

All molecular dynamics were run in the following workflow, all at 300 K and 1 bar. When the simulation required them the Berendsen Thermostat and Berendsen Barostat were used. The parameters for them were τ_T = 0.1 ps, τ_P = 5 ps, compressibility = 4.5×10^{-5} bar^{-1} where τ_T is the time constant for the thermal coupling to the bath and τ_P is the time constant for the coupling to the barostat.

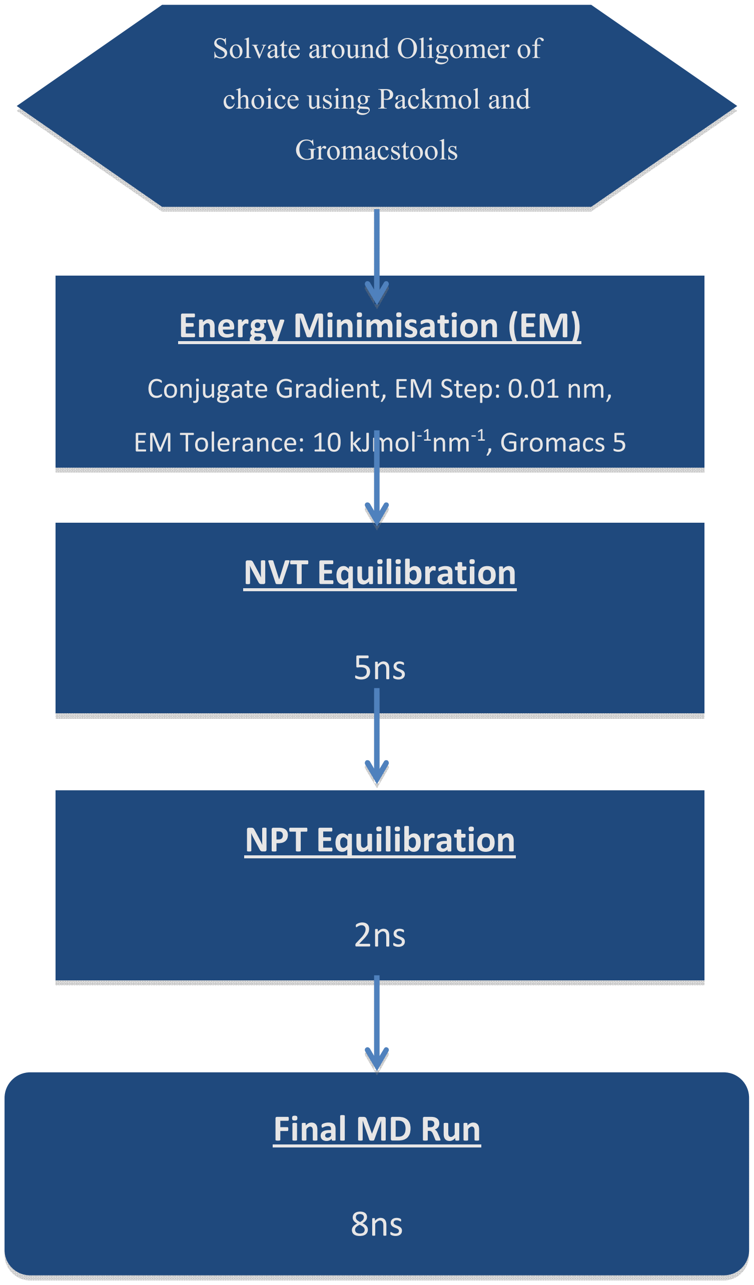
Solvate around Oligomer of choice using Packmol and Gromacstools
Energy Minimisation (EM)
Conjugate Gradient, EM Step: 0.01 nm,
EM Tolerance: 10 kJmol-1nm-1, Gromacs 5
NVT Equilibration
5ns
NPT Equilibration
2ns
Final MD Run
8ns

4. Forcefields

The basis forcefield chosen for molecular dynamics was OPLS-AA.[13,14] We then chose atom types within the OPLS set that most closely matches the local chemistry of the atoms in the oligomers of study. Carbon atoms in the phenyl groups were chosen to be equivalent to benzene ring carbons. The bridging carbon was chosen to be equivalent to the core carbon of neopentane. Sulfur and oxygen in P7 and P10 were chosen from the sulfone group in OPLS. All bond and angle utilized OPLS forces for those atom types with the average bond distance and angle taken from the DFT calculations performed. Dihedral interactions were fitted to DFT calculations. The water forcefield used was TIP4P,[15] methanol was the standard OPLS model for methanol.[4]

5. Calculations of free energy of solvation and thermodynamic driving force

Computational details of the potential calculations

We calculate the adiabatic IP, EA, IP*, and EA* potentials of the oligomer model P containing twelve phenylene equivalent units of a polymer using a ΔDFT approach from the Gibbs free energy difference (ΔG_r) of the following four redox half-reactions, written, in line with convention, as reductions:

$P^+ + e^- \rightarrow P$ (1)

$P + e^- \rightarrow P^-$ (2)

$P^+ + e^- \rightarrow P^*$ (3)

$P^* + e^- \rightarrow P^-$ (4)

where P^-, P^+, and P* are the polymer with an excess electron, hole and (singlet) exciton, respectively. The calculated ΔG_r values are converted to reduction potentials E via:

$$\Delta G_r = -nFE \quad (5)$$

Here, F is the Faraday constant and n the number of electrons taking part in the half-reaction. In our calculations on oligomers, we furthermore equate ΔG_r to the total energy difference, neglecting as discussed in the text the vibrational, translational and rotational contribution to the free energy. In previous work[16] on oligomers of P1, this was found to be a generally good approximation because of the relative similarity of the structures of P^+, P^-, P*, and P.

The calculated potential values are finally converted from the vacuum scale to that of the standard hydrogen electrode (SHE) by shifting them by the experimentally obtained value of the SHE absolute

potential (SHEAP). A range of experimental SHEAP values have been proposed in the literature, something that is partly related to different possible choices for thermodynamic standard states and partly due to extra-thermodynamic assumptions. Here we use, in line with our previous work, the original IUPAC proposed value of 4.44 V.[17]

Effects of the environment the polymer particles exist in during proton reduction are described using the COSMO[18] dielectric screening model, where we neglect the outlying charge correction, and a relative dielectric permittivity of 80.1 (water) and 2.38 (TEA). P* energies were obtained by a (TD-)DFT energy minimization in vacuum, followed by a single point (TD-)DFT calculation using COSMO, where all (TD-)DFT calculations make the Tamm−Dancoff approximation[19] for reasons of stability.[20] All (TD-)DFT calculations, finally, were performed using the Turbomole 7.01 code,[21,22] the B3LYP functional,[23,24] the double-ζ DZP basis-set,[2] and the m3 medium integration grid.[22]

The relevant solution half-reactions were predicted using a similar computational approach and setup as the polymer potentials, other than that in this case the vibrational, translational and rotational contribution to the free energy are not neglected. In contrast to previous work, protons are not modelled as isolated species but rather as adducts with TEA ($TEAH^+$), allowing us to predict potentials in other media than water.

All free energy of solution species finally include a standard state correction:

$$G_{corr} = RT\ln(CRT) \qquad (6)$$

Where R is the gas constant, T the temperature (293.15 K) and relevant C the standard state concentration; 1 mol L^{-1} for all solutes and 55.4 mol L^{-1} for water and 7.2 mol L^{-1} for TEA respectively when acting as the solvent.

Supplementary references

1. Sprick, R. S. *et al.* Visible-light-driven hydrogen evolution using planarized conjugated polymer photocatalysts. *Angew. Chem. Int. Ed.* **55,** 1792–1796 (2016).

2. Schäfer, A., Horn, H. & Ahlrichs, R. Fully optimized contracted Gaussian basis sets for atoms Li to Kr. *J. Chem. Phys.* **97,** 2571–2577 (1992).

3. Cossi, M., Barone, V., Cammi, R. & Tomasi, J. Ab initio study of solvated molecules: A new implementation of the polarizable continuum model. *Chem. Phys. Lett.* **255,** 327–335 (1996).

4. Rizzo, R. C. & Jorgensen, W. L. OPLS All-Atom Model for Amines: Resolution of the Amine Hydration Problem. *J. Am. Chem. Soc.* **121,** 4827–4836 (1999).

5. Marcon, V., van der Vegt, N., Wegner, G. & Raos, G. Modeling of Molecular Packing and Conformation in Oligofluorenes. *J. Phys. Chem. B* **110,** 5253–5261 (2006).

6. Wildman, J., Repiščák, P., Paterson, M. J. & Galbraith, I. General Force-Field Parametrization Scheme for Molecular Dynamics Simulations of Conjugated Materials in Solution. *J. Chem. Theory Comput.* **12,** 3813–3824 (2016).

7. Malvern Instruments Ltd & Instruments, M. Dynamic light scattering: An introduction in 30 minutes. *Http://Www.Malvern.Com/En/Products/Technology/Dynamic-Light-Scattering/* 1–8 (2000).

8. Bryant, G. & Thomas, J. C. Improved Particle Size Distribution Measurements Using Multiangle Dynamic Light Scattering. *Langmuir* **11,** 2480–2485 (1995).

9. Berendsen, H. J. C., van der Spoel, D. & van Drunen, R. GROMACS: A message-passing parallel molecular dynamics implementation. *Comput. Phys. Commun.* **91,** 43–56 (1995).

10. Lindahl, E., Hess, B. & van der Spoel, D. GROMACS 3.0: a package for molecular simulation and trajectory analysis. *J. Mol. Model.* **7,** 306–317 (2001).

11. Van Der Spoel, D. *et al.* GROMACS: Fast, flexible, and free. *J. Comput. Chem.* **26,** 1701–1718 (2005).

12. Hess, B., Kutzner, C., van der Spoel, D. & Lindahl, E. GROMACS 4: Algorithms for Highly Efficient, Load-Balanced, and Scalable Molecular Simulation. *J. Chem. Theory Comput.* **4,** 435–447 (2008).

13. Caleman, C. *et al.* Force Field Benchmark of Organic Liquids: Density, Enthalpy of Vaporization, Heat Capacities, Surface Tension, Isothermal Compressibility, Volumetric Expansion Coefficient, and Dielectric Constant. *J. Chem. Theory Comput.* **8,** 61–74 (2012).

14. Jorgensen, W. L., Maxwell, D. S. & Tirado-Rives, J. Development and Testing of the OPLS All-Atom Force Field on Conformational Energetics and Properties of Organic Liquids. *J. Am. Chem. Soc.* **118,** 11225–11236 (1996).

15. Jorgensen, W. L., Chandrasekhar, J., Madura, J. D., Impey, R. W. & Klein, M. L. Comparison of simple potential functions for simulating liquid water. *J. Chem. Phys.* **79,** 926–935 (1983).

16. Guiglion, P., Butchosa, C. & Zwijnenburg, M. A. Polymeric watersplitting photocatalysts; a computational perspective on the water oxidation conundrum. *J. Mater. Chem. A* **2,** 11996–12004 (2014).

17. Trasatti, S. The absolute electrode potential: an explanatory note (Recommendations 1986). *Pure Appl. Chem.* **58,** 955–966 (1986).

18. Klamt, A. & Schüürmann, G. COSMO: a new approach to dielectric screening in solvents with explicit expressions for the screening energy and its gradient. *J. Chem. Soc., Perkin Trans. 2* **0,** 799–805 (1993).

19. Hirata, S. & Head-Gordon, M. Time-dependent density functional theory within the Tamm–Dancoff approximation. *Chem. Phys. Lett.* **314,** 291–299 (1999).

20. Peach, M. J. G., Williamson, M. J. & Tozer, D. J. Influence of Triplet Instabilities in TDDFT. *J. Chem. Theory Comput.* **7,** 3578–3585 (2011).

21. Ahlrichs, R., Bär, M., Häser, M., Horn, H. & Kölmel, C. Electronic structure calculations on workstation computers: The program system Turbomole. *Chem. Phys. Lett.* **162,** 165–169 (1989).

22. Furche, F. *et al.* Turbomole. *Wiley Interdiscip. Rev. Comput. Mol. Sci.* **4,** 91–100 (2014).

23. Becke, A. D. Density-functional thermochemistry. III. The role of exact exchange. *J. Chem. Phys.* **98,** 5648–5652 (1993).

24. Stephens, P. J., Devlin, F. J., Chabalowski, C. F. & Frisch, M. J. Ab Initio Calculation of Vibrational Absorption and Circular Dichroism Spectra Using Density Functional Force Fields. *J. Phys. Chem.* **98,** 11623–11627 (1994).
