# Supplementary material for: Understanding structure-activity relationships in linear polymer photocatalysts for hydrogen evolution
Source: Nat Commun. 2018 Nov 23;9:4968. doi: 10.1038/s41467-018-07420-6 (PMC6251929; doi:10.1038/s41467-018-07420-6)
Supplement: Supplementary file 2 — Description of Additional Supplementary Files [file 41467_2018_7420_MOESM2_ESM.docx]

**Description of Additional Supplementary Files**

**File Name**: Supplementary Data 1

**Description**: DFT total energies of relevant structures used to calculate potentials.

**File Name**: Supplementary Data 2

**Description**: Xyz coordinates of P10_2 oligomer model with excess electron optimised in TEA (P-).

**File Name**: Supplementary Data 3

**Description**: Xyz coordinates of P10_2 oligomer model with excess electron optimised in water (P-).

**File Name**: Supplementary Data 4

**Description**: Xyz coordinates of P10_2 oligomer model with excess hole optimised in TEA (P+).

**File Name:** Supplementary Data 5

**Description**: Xyz coordinates of P10_2 oligomer model with excess hole optimised in water (P+).

**File name**: Supplementary Data 6

**Description**: Xyz coordinates of S1 excited state of P10_2 oligomer model optimised in water (P*).

**File Name**: Supplementary Data 7

**Description**: Xyz coordinates of P10_2 oligomer model optimised in TEA (P).

**File Name**: Supplementary Data 8

**Description**: Xyz coordinates of P10_2 oligomer model optimised in water (P).

**File Name**: Supplementary Data 9

**Description**: Xyz coordinates of P10 oligomer model with excess electron optimised in TEA (P-).

**File Name**: Supplementary Data 10

**Description**: Xyz coordinates of P10 oligomer model with excess electron optimised in water (P-).

**File Name**: Supplementary Data 11

**Description**: Xyz coordinates of P10 oligomer model with excess hole optimised in TEA (P+).

**File Name**: Supplementary Data 12

**Description**: Xyz coordinates of P10 oligomer model with excess hole optimised in water (P+).

**File Name**: Supplementary Data 13

**Description**: Xyz coordinates of S1 excited state of P10 oligomer model optimised in water (P*).

**File Name**: Supplementary Data 14

**Description**: Xyz coordinates of P10 oligomer model optimised in TEA (P).

**File Name**: Supplementary Data 15

**Description**: Xyz coordinates of P10 oligomer model optimised in water (P).
